# Choroid plexus defects in Down syndrome brain organoids enhance neurotropism of SARS-CoV-2

**DOI:** 10.1126/sciadv.adj4735

**Published:** 2024-06-05

**Authors:** Mohammed R. Shaker, Andrii Slonchak, Bahaa Al-mhanawi, Sean D. Morrison, Julian D. J. Sng, Justin Cooper-White, Alexander A. Khromykh, Ernst J. Wolvetang

**Affiliations:** ^1^ Australian Institute for Bioengineering and Nanotechnology, The University of Queensland, Brisbane, Queensland 4072, Australia.; ^2^ UQ Centre in Stem Cell Engineering and Regenerative Engineering (UQ StemCARE), The University of Queensland, Brisbane, Queensland 4072, Australia.; ^3^ School of Chemistry and Molecular Biosciences, The University of Queensland, St. Lucia, Brisbane, Queensland 4072, Australia.; ^4^ School of Chemical Engineering, The University of Queensland, Brisbane, Queensland 4072, Australia.; ^5^ GVN Centre of Excellence, Australian Infectious Diseases Research Centre, Brisbane, Queensland, Australia.

## Abstract

Why individuals with Down syndrome (DS) are more susceptible to SARS-CoV-2–induced neuropathology remains elusive. Choroid plexus (ChP) plays critical roles in barrier function and immune response modulation and expresses the ACE2 receptor and the chromosome 21–encoded TMPRSS2 protease, suggesting its substantial role in establishing SARS-CoV-2 infection in the brain. To explore this, we established brain organoids from DS and isogenic euploid iPSC that consist of a core of functional cortical neurons surrounded by a functional ChP-like epithelium (ChPCOs). DS-ChPCOs recapitulated abnormal DS cortical development and revealed defects in ciliogenesis and epithelial cell polarity in ChP-like epithelium. We then demonstrated that the ChP-like epithelium facilitates infection and replication of SARS-CoV-2 in cortical neurons and that this is increased in DS. Inhibiting TMPRSS2 and furin activity reduced viral replication in DS-ChPCOs to euploid levels. This model enables dissection of the role of ChP in neurotropic virus infection and euploid forebrain development and permits screening of therapeutics for SARS-CoV-2–induced neuropathogenesis.

## Teaser

Brain organoids with ChP-like epithelium show that Down syndrome ChP defects promote SARS-CoV-2 neuronal infection.

## INTRODUCTION

The choroid plexus (ChP) is a highly vascularized secretory tissue located within each ventricle of the vertebrate brain (*1*). ChP supports the central nervous system (CNS) by producing up to 500 ml of cerebrospinal fluid (CSF) per day in the adult human brain (*1*), while preventing infiltration of immune cells into the CNS (*1*), and during development produces a variety of signaling factors that orchestrate cortical development and neurogenesis. During early development, the lateral ventricle (LV) ChP (LVChP) anlage and the directly adjacent cortical hem, an important brain organizer region, are co-specified and both secrete and respond to morphogens such as Notch, WNT, and bone morphogenetic protein (BMP) (*2*). This developmental path ensures that ChP is always in close vicinity to the cerebral cortex and this anatomical juxtaposition between ChP and the cortex persists throughout life (*3*). Human ChPs in LV, 3V, and 4V are transcriptionally heterogeneous. The human 4VChP appears to complete differentiation earlier than the other ChPs (*4*). The human ChP acquires barrier, secretory, and transport capacities after 2 weeks of development via acquisition of tight junctions, ion channels (e.g., K^+^ voltage-gated channels), and transporters, which change over development (*5*). To date, mammalian ChP development has been predominantly studied in animal models (*6*–*8*), and it remains largely unclear to what extent these developmental processes are conserved in human. Human induced pluripotent stem cell (hiPSC)–derived three-dimensional (3D) models provide opportunities to interrogate the roles of ChP in development and diseases such neurodegeneration. To model the human ChP, several 3D organoid models have been developed, which allow the generation of ChP-like structures in vitro starting from human embryonic stem cells (hESC) (*9*, *10*). Both of these ChP organoid models exhibited maturation over time to become progressively similar to adult human ChP tissues. The ChP organoid model developed by Pellegrini *et al*. (*9*) reported the presence of neural progenitors and neurons on day 46, which, in contrast to telencephalic organoids, was progressively reduced as the ChP organoids matured. These ChP organoid models were instrumental in studying the impact of severe acute respiratory syndrome coronavirus 2 (SARS-CoV-2) infection on human brain (*10*, *11*). For instance, it was demonstrated that SARS-CoV-2 productively infects and damages the ChP epithelium, causing leakage of this brain barrier (*11*), and the authors next showed that ChP organoids infected with SARS-CoV-2 exhibited elevation of cell death and up-regulation of inflammatory genes (*10*).

Down syndrome (DS) is a genomic disorder with an incidence of 1 in 700 to 1000 live births (*12*), which is caused by the presence of a supernumerary chromosome 21 (HSA21). The extra copy of HSA21 (trisomy 21) results in neuropathological changes such as disorganized cortical lamination (*13*), altered cerebellar organization and function (*14*), and a hypocellular hippocampal dentate gyrus (*15*). The DS cerebral cortex further exhibits a reduction in excitatory neurons (*16*), an increased production of astrocytes and inhibitory neurons (*17*), as well as defective oligodendrocyte differentiation and myelination (*18*). The developing DS brain and a DS mouse model (*19*) further display ventriculomegaly that was linked to increased dosage of the HSA21 genes *PCNT* and *PCP4* involved in cilia function, and defective cilia in human DS fibroblast cells were previously reported (*20*). Cerebral organoids derived from DS iPSC were previously found to recapitulate various aspects of altered DS brain development (*21*), including defective generation of cortical neurons (*22*) and Alzheimer’s disease (AD)–like pathology (*23*). Accumulating clinical evidence indicates that SARS-CoV-2 infection of people with DS is associated with a 4-fold increased risk in hospitalization and a 10-fold increased risk of death as compared to euploid counterparts that cannot be readily explained by comorbidities (*24*, *25*). It was hypothesized that increased susceptibility to COVID-19 pathology may in part be explained by an exaggerated interferon (IFN) response brought about by the increased gene dosage of IFN pathway genes located on HSA21, and in part by defects in systemic immune system function (*26*, *27*) previously linked to increased bacterial and viral infections in people with DS (*28*–*30*). It is also possible that the increased dosage of transmembrane serine protease 2 (TMPRSS2), an HSA21 gene that codes for a protease that promotes interaction between SARS-CoV-2 spike protein and the angiotensin-converting enzyme 2 (ACE2) receptor (*31*), plays a role. Neurotropism of SARS-CoV-2 is increasingly recognized as a possible driver of long-term cognitive and sensory impairment (Long Covid) (*32*). However, since people with DS intrinsically exhibit a range of progressive interindividually highly variable cognitive deficits and a dramatically increased risk of early-onset Alzheimer’s-like disease, the long-term impact of SARS-CoV-2 infection on the cognitive function of individuals with DS is difficult to quantify. It also remains to be determined to what extent vertical transmission of SARS-CoV-2 from mother to fetus can interfere with brain development, and whether this more severely affects DS brain development.

Here, we report the generation of human cortical brain organoids that both are surrounded by a functional ChP-like epithelium and contain developing cortical cell types (ChPCOs). We demonstrate that these ChPCOs display typical neuropathological changes of DS such as reduction in oligodendrocyte progenitor cells (OPCs). We further discovered that DS ChPCOs exhibit aberrant ciliogenesis and defective polarity of the ChP-like epithelium. Strikingly, we show that the ChP-like epithelial compartment of ChPCOs strongly facilitates the neuroinvasion and neurotropism of SARS-CoV-2 and that this is increased in DS. We further show that treatments with US Food and Drug Administration (FDA)–approved drugs that inhibit TMPRSS2 activity as well as a the furin inhibitor and remdesivir reduce SARS-CoV-2 replication in DS organoids to a level comparable to the euploid group, suggesting that increased gene dosage of TMPRSS2 in the DS ChP-like epithelium may be involved, and indicating that ChPCOs are a suitable model for identifying drugs that can reduce the impact of SARS-CoV-2 on the mature and developing CNS.

## RESULTS

### Self-assembly of ChP organoids recapitulates embryonic development

**Fig. 1.**
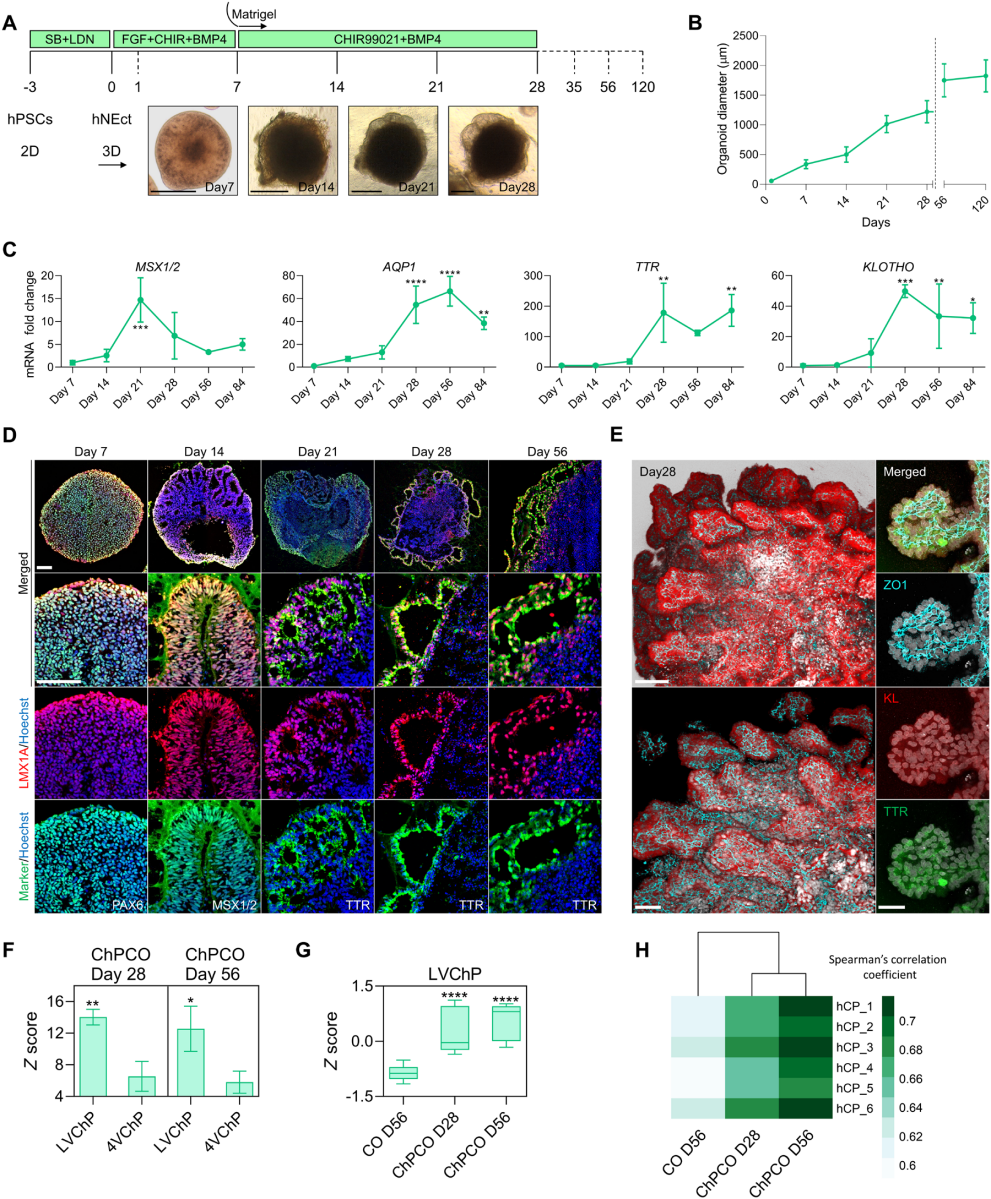
Generation of human self-organizing multiple ChPCOs in 3D from neuroectoderm. (**A**) Schematic representation of the strategy used to generate ChPCOs from hPSCs. Below brightfield images showing the developmental stages of ChPCO overtime in vitro. Scale bar, 500 μm. (**B**) Graph showing ChPCO growth in vitro. Data are presented as means ± SD. *N* = 13; total organoids *N* = 156 summarized in table S8. (**C**) qRT-PCR of *MSX1/2*, *AQP1*, *TTR*, and *KLOTHO*. Values were normalized to GAPDH levels and expressed relative to day 7 values. Data are means ± SD; *n* = 4. *P < 0.05, **P < 0.01, ***P < 0.001, ****P < 0.0001 via one-way analysis of variance (ANOVA). (**D**) Immunostaining of section organoids showing the protein expression of PAX6 (green), LMX1A (red), MSX1/2 (green), LMX1A (red), TTR (green), and LMX1A (red) and counterstained with Hoechst 33342 (blue). Scale bar, 72 μm. (**E**) Whole-mount immunostaining of ChPCO on day 42 of differentiation. Left images showing the multiple ChP-like epithelia stained with KLOTHO (red) and ZO1 (cyan), Scale bar, 110 μm. Right images are 100× magnification of a single ChP-like epithelium stained with ZO1 (cyan), KLOTHO (red), and TTR (green), Scale bar, 30 μm. (**F**) Graph representing the average *z* score per column (pool of three replicates), showing distribution of genes for LVChP and 4VChP obtained from bulk RNA-seq. Data are presented as means ± SD. *P < 0.05, **P < 0.01 via Student’s *t* test. (**G**) Box blot showing distribution of marker genes for LVChP obtained from bulk RNA-seq of ChPCOs and COs. Data are presented as minimum to maximum. ****P < 0.0001 via one-way ANOVA. See fig. S2E for individual genes used. (**H**) Heatmap comparing the Spearman correlation coefficient of the bulk RNA-seq of ChPCOs and COs to adult human ChP obtained from 44- to 70-year-old donors (*104*).

Human neuroectodermal (hNEct) cells are primed to exclusively develop into tissues of the anterior body (*33*) and are fated to form the cortex dorsally and the cortical hem ventrally, which next differentiates into ChP. We therefore reasoned that hNEct cells would be an appropriate starting population for the generation of self-organizing cortical organoids (COs) surrounded by ventricular structures derived from the ChP-like epithelium (here termed ChPCOs). We exposed human ES and iPSC lines (fig. S1A) (H9 (*34*), WTC (*35*), and G22 (*35*) to dual SMAD inhibition with SB and LDN for 3 days, which resulted in the efficient generation of hNEct cells that generate neural stem cells characterized by the expression of SOX2 and NESTIN (fig. S1B). hNEct cells were next lifted to form 3D aggregates on ultralow attachment six-well plates in N2 medium supplemented with basic fibroblast growth factor (bFGF), resulting in the formation of spheres with a neuroepithelial layer (Fig. 1A, day 7) and multiple SOX2-expressing rosettes at the center (fig. S1C). To mimic the secretion of BMP4 and WNTs by the cortical hem that specifies the dorsal cortical hem into ChP in vivo (*36*), we treated these spheres with BMP4 and CHIR99021 to promote ChP formation (Fig. 1A and fig. S1C). Since the amount of BMP4 signaling is known to promote ChP lineage differentiation at the expense of neural lineages (*9*, *10*), we exposed hNEct to increasing BMP4 dosages during the initial 4 days of the ChPCO protocol. Low doses of BMP4 (2.5 ng) reduced the proportion of SOX2-expressing cells in the early organoids to 50% in an outside-inside fashion and were further adopted as the optimal concentration (fig. S1D), and increasing the dosage to 50 ng/ml BMP4 further reduced the proportion of SOX2-expressing cells, as expected (fig. S1D). Quantification of mRNA levels of cortical hem (*MSX1/2*) and ChP genes (*AQP1*, *TTR*, and *KLOTHO*) by quantitative polymerase chain reaction (qPCR) revealed that 21 days of CHIR99021 and BMP4 treatment resulted in a significant induction of *MSX1/2* expression followed by a sharp reduction from day 28 to day 84 (Fig. 1C), suggesting the induction of cortical hem. In agreement with these data, cross-sectioned organoids revealed coexpression of cortical hem markers MSX1/2 and LMX1A proteins in the folded epithelial cells by day 14 (Fig. 1D). Between day 21 and day 28, we detected the emergence of thin epithelial layers surrounding the organoid (Fig. 1A), and qPCR demonstrated the concomitant up-regulation of ChP markers, *AQP1*, *TTR*, and *KLOTHO* (Fig. 1C). Consistent with these observations, immunostaining revealed the expression of the definitive ChP markers TTR and LMX1A proteins in these epithelial layers (Fig. 1D). Organoid size gradually increased over the first 28 days (Fig. 1B), until reaching a mean core diameter size of 1.2 mm (Fig. 1B), and a final diameter of 1.9 mm on days 56 and 120 (Fig. 1B). High-resolution 3D imaging identified multiple ChP-like epithelia emerging from a single organoid that formed an intact epithelial covering of the entire organoid as indicated by the tight junction marker ZO1 (Fig. 1E). These cells also robustly expressed KLOTHO, an anti-aging protein known to be expressed in mouse ChP (*34*), confirming that KLOTHO expression in the ChP-like epithelium is evolutionary conserved and suggesting that its role in these cells can be studied with these human ChP organoids. To exemplify the reproducibility of the system, we generated ChPCOs from different hiPSC lines (fig. S2, A and B) and found that more than 77% of the organoids exhibited the characteristic thin TTR^+^ epithelial layers enveloping the organoid, independent of cell line, clone, or batch (fig. S2C). We further found that these organoids can survive for prolonged periods (currently 1 year in culture) without obvious signs of deterioration. Collectively, our data demonstrate that this protocol recapitulates the in vivo developmental stages of cortical hem patterning and ChP-like epithelium formation and outlines a rapid protocol for generating human brain organoids that are encased in ChP-like epithelium.

In an effort to further simplify the protocol, we attempted to generate ChPCOs without embedding hNEct spheres into the Matrigel. However, non-embedded organoids treated with BMP4 and CHIR99021 for 28 days failed to generate the thin epithelial layers surrounding the organoids and led to substantial amounts of cell death in the culture (fig. S1E), indicating that extracellular matrix (ECM) plays a critical role in ChP morphogenesis in vitro.

We next compared the bulk RNA transcriptomes of day 28 and 56 ChPCOs with (non-BMP4 and CHIR99021-treated) COs. This revealed that the expression of cortical hem and ChP markers in day 28 and 56 ChPCOs generally increased over time, suggesting that the development of ChP-like epithelium is progressing beyond 28 days of in vitro culture, and was significantly higher than those observed in COs on day 56 (fig. S2D). These data are in agreement with our observation that the ChP-like epithelium in day 56 ChPCOs consists of thin folded epithelia with extensions of cuboidal epithelial cells expressing ChP markers (TTR^+^ and LMX1A^+^) (Fig. 1D), a characteristic observed in human fetal ChP epithelia (*1*). In vivo, BMP and sonic hedgehog (SHH) gradients pattern the LVChP and 4VChP along the dorsal axis of the neural tube, resulting in ChPs with distinct transcriptome profiles (*37*). On days 28 and 56, our ChPCOs showed a significant enrichment for genes related to LVChP rather than 4VChP (Fig. 1F and fig. S2E), in agreement with the fact that our protocol involves treatment with BMP4 without addition of SHH, and these genes were again expressed at higher levels in ChPCOs than in day 56 COs (Fig. 1G). At a whole transcriptome level, ChPCOs, but not COs, showed a high correlation to adult human ChP tissue (Fig. 1H and fig. S2F), consistent with previously published ChP organoid models (*9*, *10*).

### Progressive maturation of ChP-like epithelia in ChPCOs

**Fig. 2.**
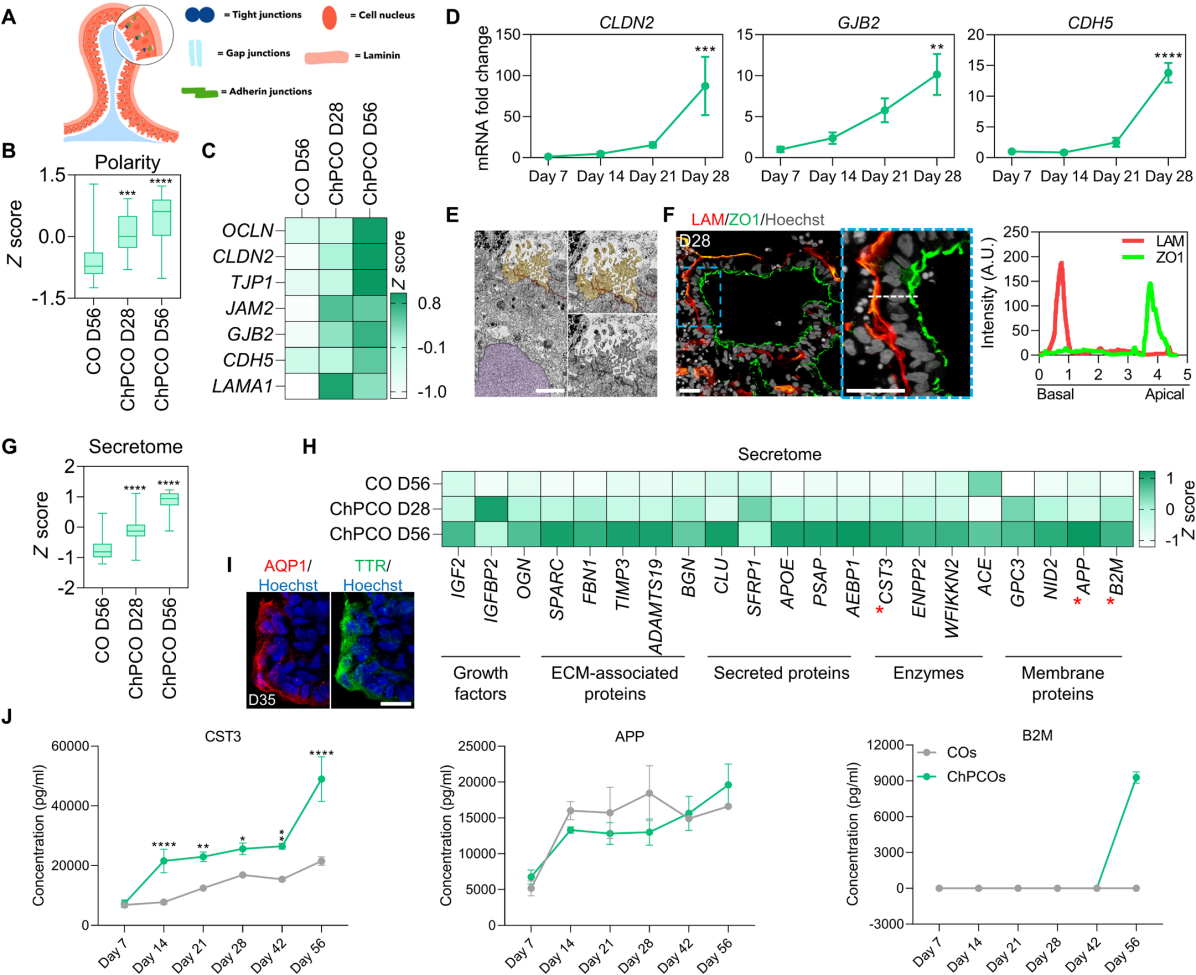
Development and functional analysis of ChP-like epithelium in ChPCOs. (**A**) Schematic diagram outlining the apicobasal polarity of ChP. (**B**) Box blot showing distribution of polarity genes [listed in (C) and fig. S3A] obtained from bulk RNA-seq. Data are minimum to maximum. ***P < 0.001, ****P < 0.0001 via one-way ANOVA. (**C**) Heatmap of representative apicobasal polarity genes within bulk RNA-seq. Values are shown as *z* score. (**D**) qRT-PCR of apicobasal polarity genes in ChPCOs. All values were normalized to GAPDH and expressed relative to day 7 values. Data are means ± SD. **P < 0.01, ***P < 0.001, ****P < 0.0001 via one-way ANOVA. *N* = 3. (**E**) TEM of ChPCOs on day 28 showing the high density of mitochondria, tight junction at the apical side, microvilli (light brown), and nucleus (light purple). Scale bar, 250 nm. (**F**) ChPCO sections stained with LAMININ (red) and protein ZO1 (green) and counterstained with Hoechst 33342. Scale bar, 20 μm. Dotted white line represents the average intensity of LAMININ and ZO1 expression along the apicobasal of ChP-like epithelium plotted in the graph. (**G**) Box blot showing distribution of CSF secretome genes [listed in (H)] obtained from bulk RNA-seq. Data are presented as minimum to maximum. ****P < 0.0001 via one-way ANOVA. (**H**) Heatmap of CSF secretome genes obtained from bulk RNA-seq. Values are *z* score. Red asterisks represent the genes selected for ELISA experiment shown in (J). (**I**) ChPCO sections immunostained with water channel aquaporin 1 (AQP1) in red and TTR in green in ChP-like epithelium and counterstained with Hoechst 33342. Scale bar, 20 μm. (**J**) Luminex multiplex/ELISA showing CSF secretome protein markers in medium of ChPCOs. Data are shown as means ± SD; *N* = 3. *P < 0.05, **P < 0.01, ****P < 0.0001 via one-way ANOVA.

To determine whether these self-organized ChP-like epithelial layers in ChPCOs exhibit similar properties to the in vivo ChP, we next examined the establishment of epithelial polarity, abundance of mitochondria, ciliogenesis, and CSF secretion (*38*). We first assessed the establishment of epithelial polarity in the ChP-like epithelium, which is governed by tight junctions, gap junctions, adherent junctions, and basal lamina (Fig. 2A). In agreement with other ChP organoid models (*9*), bulk RNA-sequencing (RNA-seq) data identified a progressive enrichment of genes associated with these structures in ChPCOs over time as compared to COs (Fig. 2, B and C, and fig. S3A). qPCR analysis revealed a gradual increase of selected tight junction genes such as *CLDN11* and *CLDN12* (fig. S3B), although *CLDN2* was only significantly increased by day 28 (Fig. 2D). Similarly, mRNA levels of the gap junction gene *GJB2* and adherent junction genes *CDH5* and *PCDH18* also gradually increased (Fig. 2D and fig. S3B), consistent with the widespread and contiguous expression of the tight junction marker ZO1 in the ChP-like epithelial layers (fig. S3C). The establishment of tight junctions is also critical for polarization of ChP into distinct basal components (*39*), and we therefore examined whether the ChP-like epithelial cells of ChPCOs would exhibit apicobasal polarity of membrane proteins critical for normal ChP epithelial cell function. Labeling ChPCOs with ZO1 and LAMININ, which accumulate on the apical and basal side of polarized ChP-like epithelium, respectively, revealed the correct formation of the basal membrane and apical polarity (Fig. 2F). We reasoned that the establishment of ChP-like epithelial polarity in ChPCOs that is required for ChP homeostasis, CSF secretion, neurotrophic factor transport, and barrier function was an indication that the ChP-like epithelia in our ChPCOs should have the potential for CSF production and effective barrier formation. RNA-seq analysis confirmed the significant enrichment of genes that code for ChP-secreted proteins in ChPCOs on day 28 compared to COs on day 56 (Fig. 2G) and revealed that their expression substantially increases as ChPCOs further develop (Fig. 2G, ChPCOs day 56). We detected expression of *IGF2*, a growth factor known to be secreted in the CSF and that stimulates ependymal neural stem cell (NSC) proliferation (*40*), ECM genes such as *SPARC* that are involved in uptake and delivery of proteins from blood to the CSF (*41*), as well as a number of enzymes, secreted proteins, and membrane proteins, which are all involved in CSF generation and secretion (Fig. 2H). Consistent with a previous study (*9*), staining showed the correct apical localization of the water channel aquaporin 1 (AQP1) in the ChP-like epithelium of ChPCOs on day 28, a protein involved in CSF secretion (Fig. 2I). We next used a multiplex enzyme-linked immunosorbent assay (ELISA) to determine the ChPCO secretome released in the culture medium. This revealed a significant increase of CST3 and B2M of ChPCOs as compared to COs (Fig. 2J). This is consistent with the ability of ChP organoids to secrete human CSF proteins (*9*). The abundance of APP secretion was, however, similar between ChPCOs and COs (Fig. 2J).

**Fig. 3.**
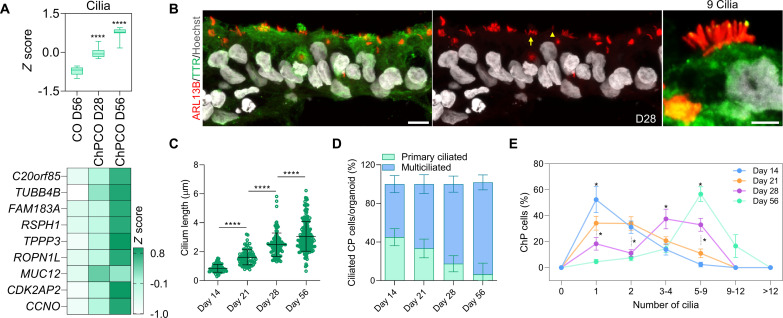
Development and functional analysis of ciliogenesis in ChP-like epithelium. (**A**) Box blot showing distribution of marker genes (listed below in the heatmap) associated with cilia obtained from bulk RNA-seq of ChPCOs on days 28 and 56 and COs on day 56. Data are presented as minimum to maximum, with notches centered on the median. ****P < 0.0001 via one-way ANOVA. Below heatmap expression of marker genes related to cilia. Values are shown as *z* score. (**B**) Analysis of immunostained ChPCO sections on day 28 showing ARL13B (red) protein expression in cilia of ChP-like epithelium marked with TTR (green). The section was counterstained with Hoechst 33342. Scale bar, 20 μm; magnified image scale bar = 5 μm. Yellow arrow indicates a multiciliated cell; yellow arrowhead indicates a mono-ciliated cell. (**C**) Quantification of cilia length in ChPCOs on days 1, 21, 28, and 56 of differentiation. Data are presented as means ± SD; ****P < 0.0001 via one-way ANOVA. Number of independent experiments = 3. Individual dots represent a cilium length. (**D**) Stacked bar graph showing the percentage of cells with a single cilium and multiple cilia in human ChP-like epithelial cells in ChPCOs on days 7, 21, 28, and 56 of differentiation. Data are presented as means ± SD. Number of independent experiments = 3. (**E**) Distribution of cilium number in ChPCOs cultured for days 14, 21, 28, and 56. The data are presented as a percentage of ChP cells with a single primary cilium or multicilia. Minimum *n* = 3 per time point. Total number of experiments = 16; total number of analyzed organoids = 40. Data are presented as means ± SD. Kolmogorov-Smirnov test, *P < 0.05. The number of organoids analyzed and the number of experiments from each time point are summarized in table S8.

Primary cilia in ChP are essential for regulating the flow and transport of CSF and can be characterized according to their length, motility, and number per cell (*42*). In vivo, ChP consists mainly of epithelial cells that are multiciliated, with tufts of cilia ranging from four to eight cilia per cells, but also contains a small fraction of ChP cells implicated in chemo- and/or osmo-sensation that extend one primary cilium into the CSF (*43*). How and when these cell types are specified in human embryos remains largely unclear. Since the generation of ChPCOs closely mimics the progressive temporal morphogenic sequence of events of normal ChP development in vivo (Fig. 1), and transmission electron microscopy (TEM) identified microvilli structure at the periphery of the ChPCOs (Fig. 2E), we examined ciliogenesis in our human ChPCOs cultured for 14, 21, 28, and 56 days. Bulk RNA-seq confirmed the significant and progressive enrichment of cilia-associated genes in ChPCOs as compared to COs (Fig. 3A). Immunostaining for cilia with the ARL13B antibody demonstrated that human ChP-like epithelial cells in ChPCOs are ciliated (Fig. 3B), and that cilia length significantly increases over time, with mean values of 0.7, 1.6, 2.3, and 3 μm on days 14, 21, 28, and 56, respectively (Fig. 3C). On day 14, the developing ChP contains almost equal amounts of mono- and multiciliated TTR-expressing cells and then displays a progressive increase in multiciliated cells to approximately 66% on day 21, 82% on day 28, and 95.4% on day 56, which is accompanied by a concomitant decrease in mono-ciliated ChP-like epithelial cells (Fig. 3D). The number of cilia on ChP-like epithelial cells in mammals has been reported to range from four to eight cilia per cell in rats to 50 cilia per cell in salamander (*1*). Our data show that human ChP-like epithelial cells in ChPCOs display a distinct shift in the number of cilia per cell over time in culture between day 14 and day 56, at which point >50% of all ChP-like epithelial cells have between five and nine cilia per cell (Fig. 3E). Bulk RNA-seq data showed no significant alteration in genes associated with cilia resorption over time (fig. S3E). Collectively, these findings indicate that the self-assembled ChPCOs recapitulate key aspects of in vivo ChP ciliogenesis, suggesting that ChPCOs may provide a useful model for investigating diseases such as DS (*19*) and Bardet-Biedl syndrome (*44*), in which defective primary cilia are thought to cause brain ventriculomegaly and hydrocephalus, respectively.

### Specialized functional cellular compartments of cortical tissue arise in ChPCOs

**Fig. 4.**
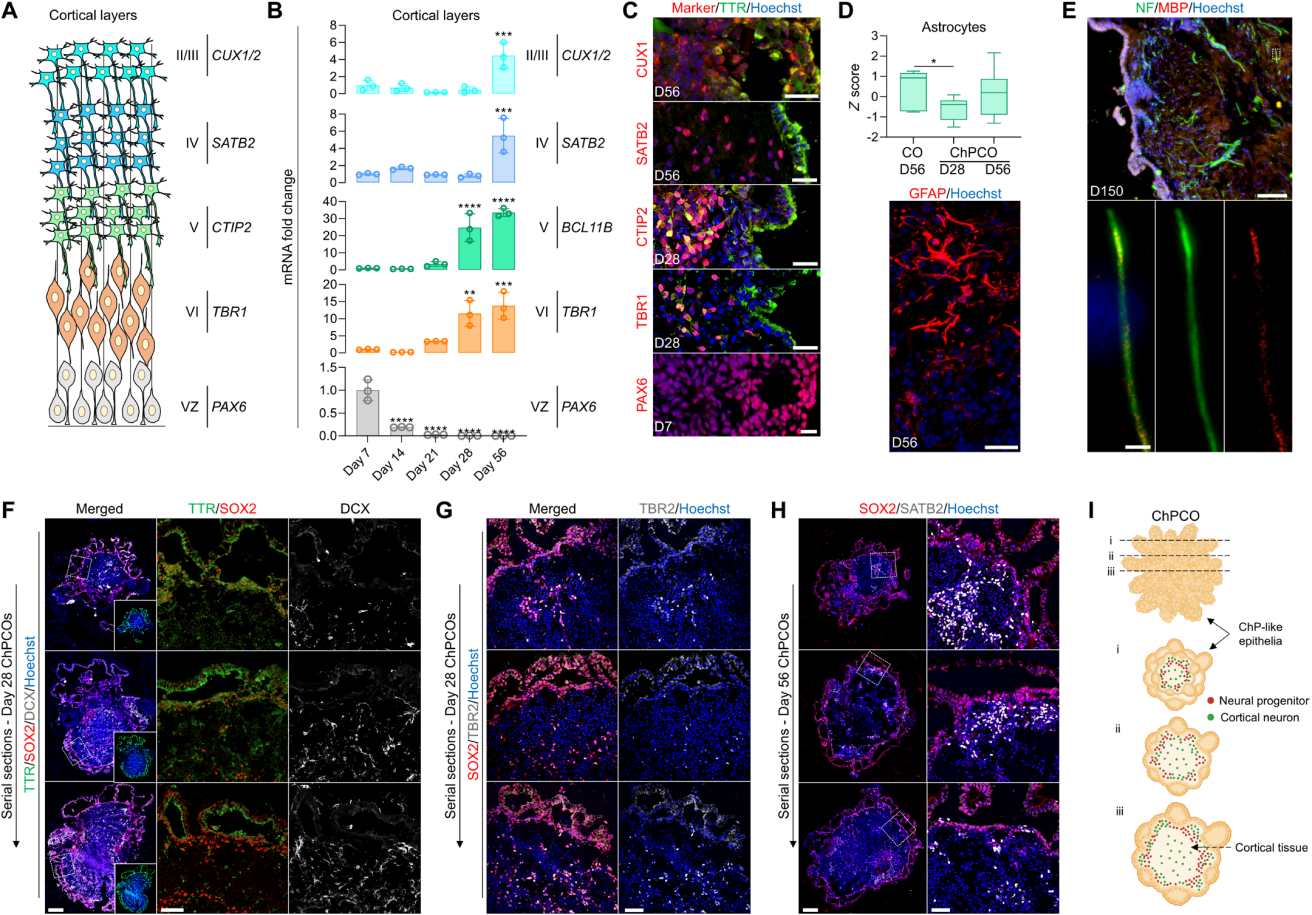
ChPCOs contain cortical neurons with progress maturation over time in culture. (**A**) Diagram of the development of the cortical plate layers: VZ is ventricle zone. (**B**) qRT-PCR of ventricular zone gene *PAX6* and cortical neuronal layer genes (*TBR1*, *CTIP2*, *SATB2*, and *CUX1*/2). All values were normalized to GAPDH and expressed relative to day 7 values. Data are means ± SD; **P < 0.01, ***P < 0.001, ****P < 0.0001 via one-way ANOVA. *N* = 3. (**C**) Immunostaining of ChPCO sections showing PAX6 (red), TBR1 (red), CTIP2 (red), SATB2 (red), and CUX1 (red) and counterstained with Hoechst 33342. Scale bars, 10 μm (PAX6), 20 μm (TBR1, CTIP2, and SATB2), and 30 μm (CUX1). (**D**) Box blot showing distribution of astrocyte genes (listed in fig. S4B) obtained from bulk RNA-seq. Data are presented as minimum to maximum. *P < 0.05 via one-way ANOVA. Below image shows ChPCO section immunostained with GFAP (red) and counterstained with Hoechst 33342. Scale bar, 30 μm. (**E**) Immunostaining of ChPCO section showing myelinated neurons marked by neurofilament (green) and MBP (red) and counterstained with Hoechst 33342 (blue). Scale bar, 100 μm. Dotted white box indicates zoomed images below. Scale bar, 2 μm. (**F**) Immunostaining of serial sections of ChPCO showing DCX (gray), SOX2 (red), and TTR (green). Scale bar, 200 μm. Dotted square white box indicates zoomed images with scale bar = 50 μm. (**G**) Immunostaining of serial sections of ChPCO showing TBR2 (gray) and SOX2 (red). Scale bar, 50 μm. (**H**) Immunostaining of serial sections of ChPCO showing SATB2 (gray) and SOX2 (red). Scale bar, 200 μm. Dotted square white box indicates zoomed images with scale bar = 50 μm. (**I**) Schematic diagram outlining the architectural structure of ChPCO with neural compartment.

In addition to the ChP components outlined above, ChPCO organoids also have other neural compartments. During embryonic development, the NEct-derived rostral neural tube gives rise to ChP in the dorsomedial telencephalon and cortical plate dorsally (*33*). We investigated whether these key cellular compartments of the cortical plate layers arise in our organoids in addition to the ChP-like epithelium (Fig. 4A) by examining the expression of different cortical neuronal markers known to be expressed in COs. We found a gradual reduction of *PAX6* mRNA over time (Fig. 4B), and a concomitant increase in layer VI, V, IV, and II/III neuronal markers as indicated by the expression of *TBR1*, *CTIP2*, *SATB2*, and *CUX1/2*, respectively (Fig. 4B). Staining for these neuronal proteins in midline sectioned organoids revealed specification of cortical TBR1^+^ and CTIP2^+^ neurons on day 28 (Fig. 4C and fig. S4A), whereas SATB2^+^ and CUX1^+^ neurons were only detected on day 56 of organoid maturation (Fig. 4C). The specification of cortical layers occurred in close vicinity to the ventricle-like structures formed by the ChP-like epithelial cells (Fig. 4C and fig. S4A). Astrocytes marked by glial fibrillary acidic protein (GFAP) were also specified in ChPCOs and COs on day 56 of differentiation (Fig. 4D), but not in ChPCOs on day 28 (fig. S4C), in agreement with the notion that astrogliogenesis commences around 50 days (*45*). Furthermore, we detected oligodendrocytes marked by PDGFRA and CNPase on day 56 (fig. S3D), and colocalization of myelin basic protein (MBP) and neurofilament was observed in the mature organoids on day 150 (Fig. 4E), suggesting the onset of myelination. To further understand the architectural structure of ChPCO model, we included serial sections of ChPCOs on days 28 (Fig. 4, F and G) and 56 (Fig. 4H), stained with SOX2, DCX, TBR2, and SATB2 to mark neural progenitors, early specified neuronal progenitors, intermediate neural progenitors in deep layers, and superficial cortical neuronal layers, respectively (Fig. 4, F to H). We found that SOX2^+^ cells are located closer to the ChP-like facing surface, while others are internal and distant from the ChP-like tissue (Fig. 4, F to I). Similarly, cortical neurons are distributed throughout the cortical tissue in ChPCOs (Fig. 4, G to I).

**Fig. 5.**
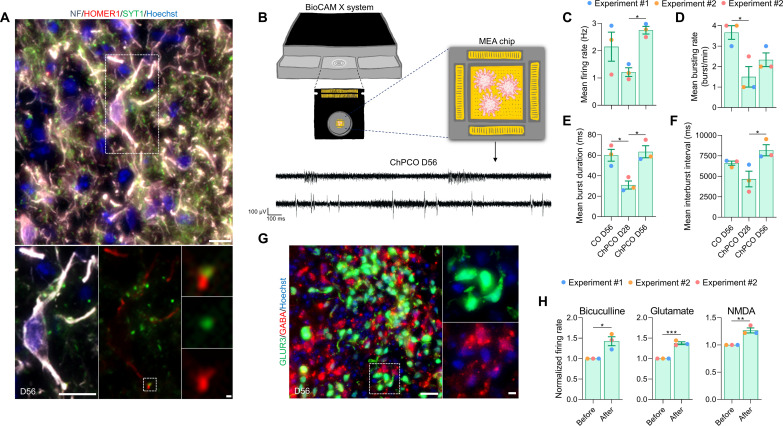
Functional analysis of cortical neurons in ChPCOs. (**A**) Analysis of immunostaining of ChPCO sections on day 56 showing neurons with post- and presynapses marked by neurofilament (gray), HOMER1 (red), and SYNT1 (green). Scale bar, 10 μm. Dotted rectangular white box indicates zoomed images of a neuron shown below with scale bar = 1 μm. Doted square white box indicates zoomed images of connected pre- and post-synapse with scale bar = 1 μm. (**B**) Schematics of extracellular recordings from ChPCOs on MEA. Below are the representative transient plots from neural activities recorded in ChPCOs on day 56. Scale bar, 100 ms (horizontal) and 100 μV (vertical). (**C** to **F**) Bar graphs show the changes in the patterns of neural activity in ChPCOs on days 28 and 56 compared to COs on day 56. Bar graphs represent means ± SD in firing rate (C), mean bursting rate (D), mean burst duration (E), and mean interburst interval (F). *P < 0.05 via one-way ANOVA. Total number of experiments = 3; total number of analyzed organoids = 9. The number of organoids analyzed and the number of experiments from each time point are summarized in table S8. (**G**) Magnified image of sectioned ChPCOs on day 56 immunostained for glutamatergic (GLUR3, green) and GABAergic (GABA, red) neurons on day 56. All sections were counterstained with Hoechst 33342 (blue). Scale bar, 20 μm; scale bar for magnified image = 10 μm. (**H**) Bar graphs showing the changes in the patterns of mean firing rate before and after drug treatments. The following drugs were used: 50 μM glutamate, 10 μM NMDAA, and 50 μM bicuculline. *P < 0.05, **P < 0.01, ***P < 0.001 via Student’s *t* test.

To evaluate the functional properties of the cortical neurons, we first confirmed that mature neurons exhibited the expression and juxtaposition of pre- and postsynaptic markers SYT1 and HOMER1, respectively (Fig. 5A), as morphological evidence of synaptic contacts. We then characterized key electrophysiological parameters using high-density multielectrode arrays (MEAs) (Fig. 5B). MEA analysis detected spontaneous electrical activity in ChPCOs from day 28 (an example of the traces on day 56 is shown in Fig. 5B). Over time, the spontaneous neural firing/bursting rates progressively increased (Fig. 5, C to F). Conspicuously, firing rates increased significantly on day 56, when GFAP^+^ cells were observed in ChPCOs (Fig. 4D). However, the burst rate did not significantly increase in ChPCOs on day 56 in contrast to COs (Fig. 5D). We speculate that this difference may be due to the different proportions of neuronal subtypes present in these two types of organoids. We noted that 56-day-old ChPCOs contained glutamatergic (GLUR3^+^) and GABAergic (GABA^+^) neurons that were located adjacent to each other (Fig. 5G), consistent with the specification of GABAergic inhibitory neurons during in vivo cortical development (*46*). Treatment with the GABA receptor antagonist bicuculline (50 μM) significantly increased the firing rate in ChPCOs (Fig. 5H and fig. S4E), confirming the presence of inhibitory synaptic transmission, while acute glutamate (200 μM) and *N*-methyl-d-aspartate (NMDA) (50 μM) treatments significantly increased the firing rate (Fig. 5H and fig. S4E), demonstrating the presence of active glutamatergic and GABAergic neurons. Collectively, these data suggest that neural networks with excitatory and inhibitory neural circuits were well established within ChPCOs on day 56.

### DS ChPCOs recapitulate key aspects of DS brain pathology

**Fig. 6.**
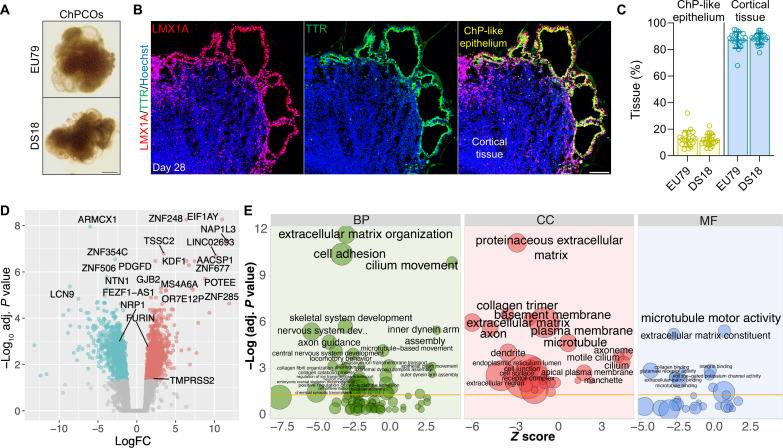
Transcriptional profile of ChPCO modeling DS. (**A**) Representative bright-field images of euploid- and DS-iPSC–derived ChPCOs on day 28. Scale bar, 500 μm. (**B**) Representative image of euploid-iPSC–derived ChPCOs on day 28 immunostained with LMX1A (red) and TTR (green). The section was counterstained with Hoechst 33342 (blue). Scale bar, 100 μm. (**C**) Quantification of ChP-like epithelium and cortical tissues compartments in at least three different euploid (EU79) and DS (DS18) hiPSC-derived ChPCOs. Individual dots represent a single organoid. Total number of experiments = 8; total number of analyzed organoids = 29. The number of organoids analyzed and the number of experiments from each time point are summarized in table S8. (**D**) Volcano plot highlighting DEGs in euploid and DS ChPCOs on day 28. Significant up-regulated genes are shown in red, and down-regulated genes are shown in cyan. Top-most DEGs as well as *TMPRSS2*, *FURIN*, and *NRP1* genes are labeled. (**E**) Gene ontology (GO) enrichment analysis of differentially expressed genes (DEGs) in DS ChPCOs compared to euploid organoids. *Z* scores indicate the cumulative increase or decrease in expression of the genes associated with each term. Size of the bubbles is proportional to the number of DEGs associated with respective GO term. BP, biological processes; CC, cellular components; MF, molecular functions.

**Fig. 7.**
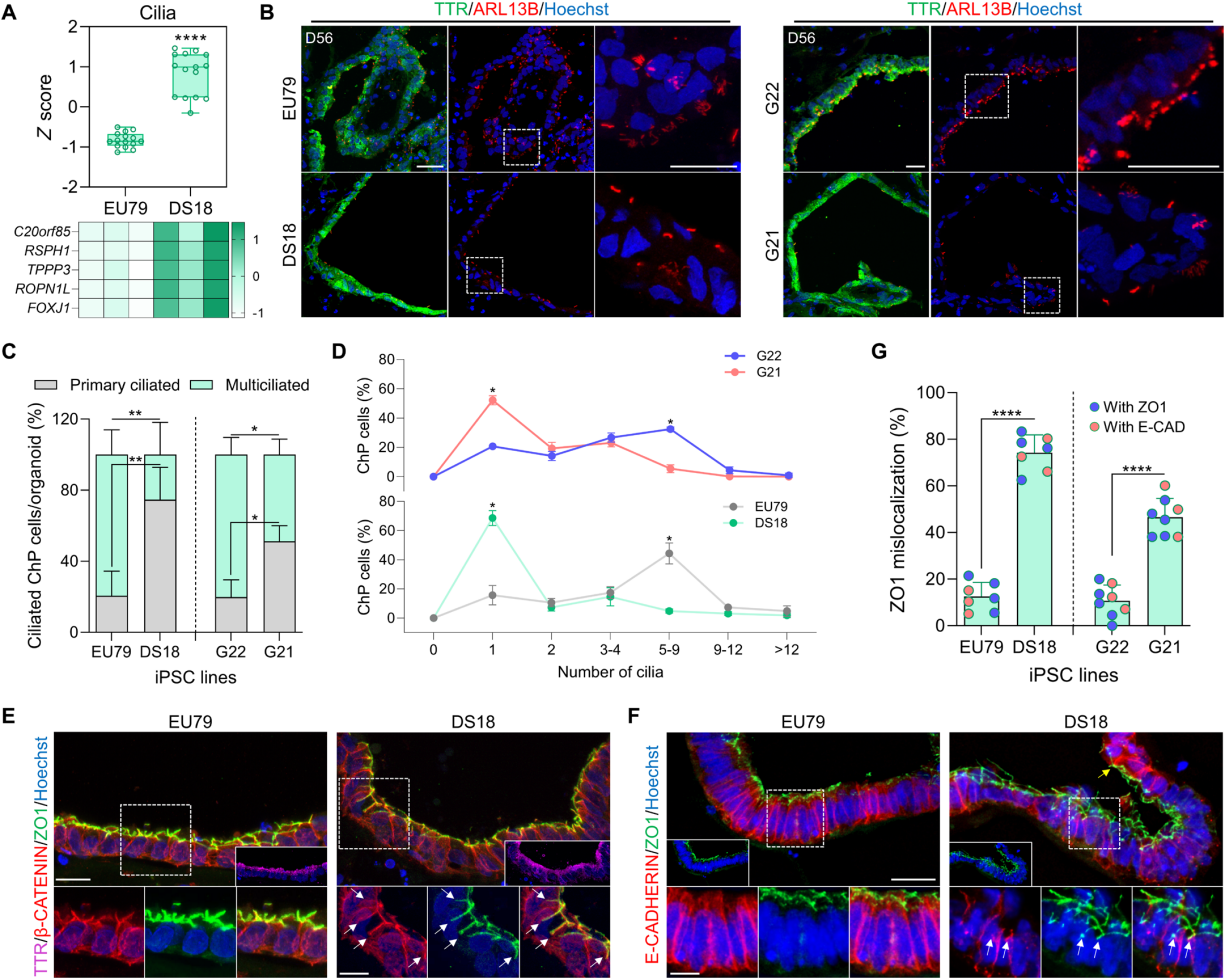
Analysis of ciliogenesis and apicobasal polarity in ChPCO derived from DS. (**A**) Box blot showing distribution of cilia genes (listed in heatmap) obtained from bulk RNA-seq of ChPCOs on day31. Data minimum to maximum. ****P < 0.0001 via one-way ANOVA. Below heatmap of cilia genes, values are shown as *z* score. (**B**) Immunostaining of day 56 ChPCOs with ARL13B (red) and TTR (green) and counterstained with Hoechst 33342. Scale bar, 20 μm. Dotted white boxes denote areas that are magnified. (**C**) Stacked bar graph demonstrating the percentage of cells with a single cilium and multiple cilia in ChP-like epithelial cells in ChPCOs. Data are means ± SD. *N* = 3. *P < 0.05; **P < 0.01 via one-way ANOVA. Total number of experiments = 13; total number of analyzed organoids = 35 as summarized in table S8. (**D**) Distribution of cilia number in ChPCOs. Total number of experiments = 14; total number of analyzed organoids = 29 summarized in table S8. Data are presented as means ± SD. Kolmogorov-Smirnov test, *P < 0.05. (**E**) Immunostaining of day 56 ChPCO sections with β-catenin (red) and ZO1 (green) and TTR (magenta) and counterstained with Hoechst 33342 (blue). Scale bar, 25 μm. Dotted white boxes denote areas that are magnified. White arrows indicate ZO1 distribution at basolateral domain. Scale bar, 10 μm. (**F**) Immunostaining of day 56 ChPCOs with E-CADHERIN (red) and ZO1 (green) and counterstained with Hoechst 33342 (blue). Scale bar, 25 μm. Dotted white boxes denote areas that are magnified. Scale bar, 10 μm. White arrows indicate ZO1 distribution at basolateral domain. Yellow arrow indicates E-CADHERIN enrichment at the apical domain. (**G**) Graph showing the percentage of mislocalized ZO1 in ChP-like epithelium of day 56 ChPCOs. Data are presented as means ± SD. ****P < 0.0001 via Student’s *t* test. *N* = 3.

DS is caused by trisomy 21 and leads to developmental delay and intellectual defects (*47*). Both the human developing DS brain and Ts65Dn DS mice further display defects in oligodendrocyte differentiation and myelination (*18*). Moreover, trisomy 21 fibroblasts exhibit a reduction in cilia formation and function (*20*), making DS brain a particularly interesting condition to model in ChPCOs. We therefore generated ChPCO organoids from an iPSC line derived from patient with DS (DS18) and its isogenic euploid counterpart, the iPSC line (EU79) (*48*) (Fig. 6A). The size of the DS ChPCO organoids was comparable to the euploid organoids (fig. S5A), suggesting similar growth rates. Quantification of the percentage of ChP-like epithelium and cortical tissue cell populations in at least three different batches of ChPCO organoids generated from the EU79 and DS18 lines revealed that ChPCOs of euploid and DS lines both reproducibly organized into ChP-like epithelium and cortical tissue regions with similar cell proportions across different batches (Fig. 6, B and C). To characterize the isogenic DS and euploid ChPCOs in more detail, we performed RNA-seq analysis (three organoids per replicate, *n* = 3) on day 28 organoids (Fig. 6D, fig. S5B, and table S1). This revealed that euploid and DS organoids exhibited distinct transcriptional profiles (fig. S5B), with 962 genes with increased expression and 997 genes with decreased expression in DS compared to the isogenic euploid organoids (Fig. 6D and table S1). Twenty-two genes (9.7%) located on HSA21 were among the top 500 differentially expressed genes (DEGs) (fig. S5D); four of these genes (*MX2*, *TMPRSS2*, *ADAMTS5*, and *RUNX1*) are associated with viral infection (fig. S5D). Enrichment analysis of up-regulated genes in DS organoids revealed enrichment in several categories, including nervous system development, cilium movement and motility, cell adhesion, and basement membrane genes (Fig. 6E). However, no changes were observed in ChP or secretome-associated genes (fig. S5C). Immunoblotting data confirmed the increase in ECM proteins that are not components of the Matrigel in DS ChPCOs as compared to the euploid organoids (fig. S6K). Notably, genes associated with ion transport were significantly up-regulated in DS organoids compared to the euploid group (fig. S6J). DS organoids displayed a significant increase in the expression of a subset of ciliogenesis markers (Fig. 7A). In contrast, immunostaining results of cilia with ARL13B antibody indicated that ChP-like epithelial cells in DS ChPCO organoids at 56 days are predominantly mono-ciliated (Fig. 7, B and C) and generated significantly less ciliated ChP-like epithelial cells than the corresponding euploid ChPCOs (Fig. 7D), although the average cilium length was comparable between the two groups (fig. S6F). Unexpectedly, we also detected a significant alteration in apicobasal polarity marker genes (fig. S6A) and proteins (fig. S6C) in DS organoids. To further investigate possible defects in cell polarity, euploid and DS organoids were stained with ZO1 and E-CADHERIN, proteins associated with apicobasal epithelial cell polarity (*49*), and β-CATENIN, a protein known to be expressed in the cell membrane of the human ChP (*50*) (Fig. 7, E and F). In euploid organoids, these proteins correctly segregated to their respective compartments in the ChP-like epithelium as expected (Fig. 7, E and F). However, in DS organoids derived from DS18 and G21 lines, ZO1 is often misplaced in the non-apical domains and colocalized with β-CATENIN at the basolateral domain (Fig. 7E and fig. S5G). Additionally, E-CADHERIN in DS ChP-like epithelium did not exhibit the same distribution as observed in the euploid group (Fig. 7F). While E-CADHERIN enriched at the most apical region and extended toward the basolateral domain in euploid ChP-like epithelium, in the DS group, substantial E-CADHERIN staining was observed in a broader domain, with notable enrichment at the apical domain overlapping with ZO1 (Fig. 7F). These observations suggest that the apicobasal polarization of ChP-like epithelia in DS organoids is disrupted. We quantified the percentage of overlap between ZO1 and β-CATENIN at the basolateral domain of ChP-like epithelial cells (*49*). We found that ZO1 colocalized at the basolateral domain in 12.5% and 12.25% of ChP-like epithelial cells in ChPCOs from the euploid EU79 and G22 lines (Fig. 7G), respectively. However, it remained colocalized at the basolateral domain in 74% and 44.7% of ChP-like epithelial cells in ChPCOs from the DS DS18 and G21 lines (Fig. 7G).

We further noted that all marker genes of oligodendrocyte precursor cells (OPCs) were down-regulated in DS organoids at 28 days (fig. S6B), and this was corroborated at the protein level via Western blotting (fig. S6C). Immunofluorescence staining of organoid sections identified clear expression of SOX10 protein in cells within the cortical tissue domain of euploid ChPCOs (fig. S6D), and quantification demonstrated a significant reduction in SOX10-expressing cells in DS ChPCOs as compared to their euploid counterparts (fig. S6E). This prompted us to examine the expression of the HSA21 gene *OLIG2* that plays a role in oligodendrogenesis in the developing dorsal forebrain (*51*), but found that this gene was underexpressed at the mRNA level in DS ChPCOs (fig. S5D). Western blot and immunofluorescence staining of organoid sections next confirmed reduced protein expression and a reduced number of cells expressing OLIG2 in DS ChPCOs as compared to euploid organoids, respectively (fig. S5, E and F). Collectively, these findings indicate that DS ChPCOs recapitulate key aspects of DS brain pathology.

### SARS-CoV-2 productively infects DS ChPCOs

**Fig. 8.**
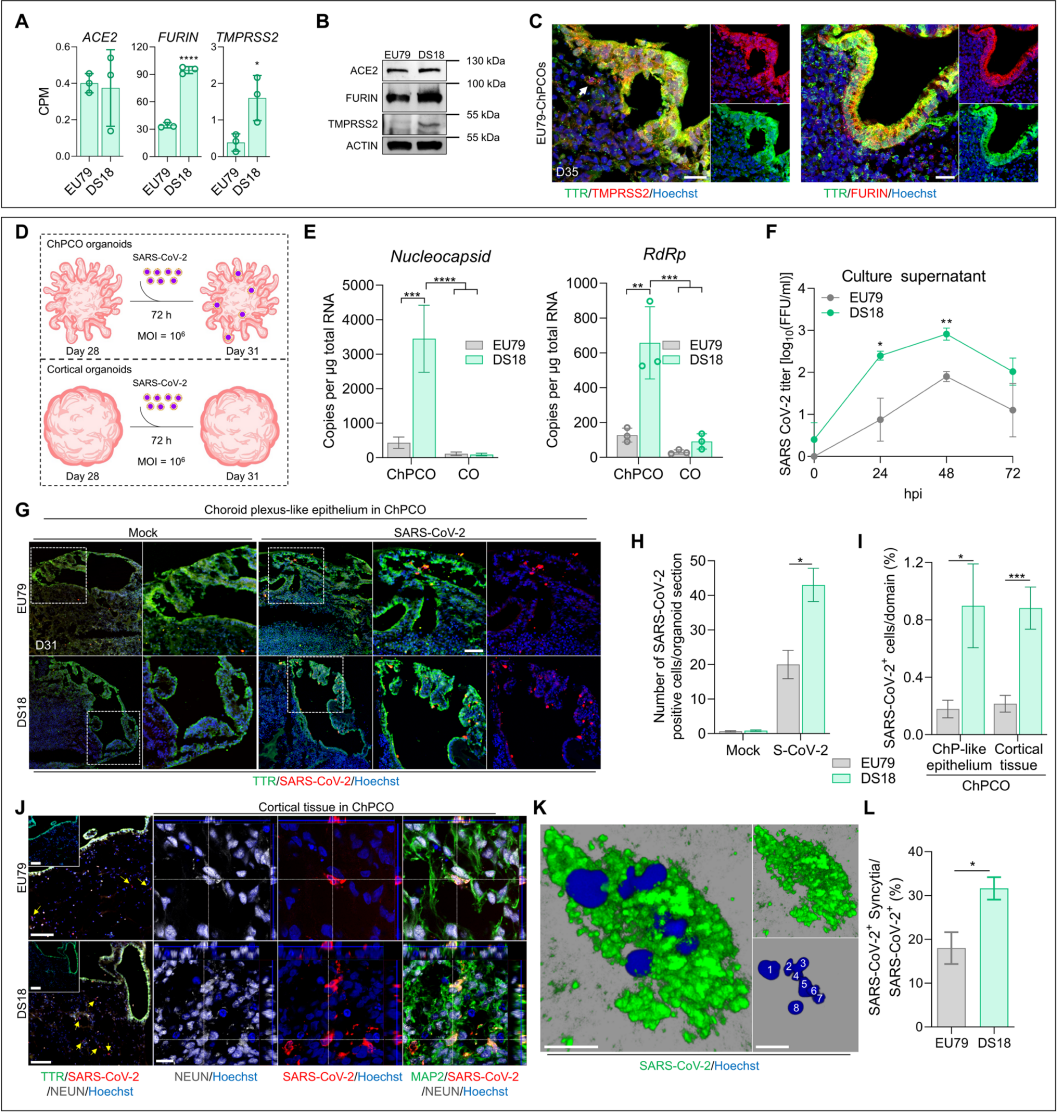
SARS-CoV-2 productively infects ChPCO but not CO organoids. (**A**) Abundance of *ACE2*-, *FURIN*-, and *TMPRSS2*-derived RNA-seq reads in day 31 ChPCOs. Data are means ± SD. *P < 0.05, ****P < 0.0001 via Student’s *t* test. *N* = 3. (**B**) Western blots showing the levels of ACE2, FURIN, TMPRSS2, and ACTIN in day 31 ChPCOs. (**C**) Section of euploid ChPCOs stained with TMPRSS2 (red), FURIN (red), and TTR (green) and counterstained with Hoechst 33342. Scale bar, 20 μm. (**D**) Schematic diagram of the SARS-CoV-2 infection protocol. (**E**) Graphs showing SARS-CoV-2 *nucleocapsid* and *RdRp* in day 31 organoids. Data are means ± SD. **P < 0.01, ***P < 0.001, ****P < 0.0001 via two-way ANOVA. *N* = 3. (**F**) Viral titers from ChPCO supernatants after SARS-CoV-2 (10^6^ FFUs) treatment. Data are means ± SEM. *N* = 3; *P < 0.05, **P < 0.01 via Student’s *t* test. (**G**) ChPCO sections stained with TTR (green) and SARS-CoV-2 spike (red) after SARS-CoV-2 (10^6^ FFUs) or mock (SARS-CoV-2 at 10^4^ FFUs) and counterstained with Hoechst 33342. Scale bar, 60 μm. (**H**) Graph showing the SARS-CoV-2–positive cells in ChPCOs. Data are means ± SEM. *P < 0.05 via Student’s *t* test. Fifteen ChPCO sections from *n* = 3. (**I**) Graph showing the percentages of SARS-CoV-2–positive cells within ChPCOs. Data are means ± SEM. *P < 0.05, ***P < 0.001 via Student’s *t* test. Fifteen ChPCO sections from *n* = 3. (**J**) ChPCO sections stained with TTR (green), SARS-CoV-2 spike (red), and NEUN (gray). Scale bar, 100 μm. Orthogonal projections are confocal z-stacks stained with MAP2 (green), SARS-CoV-2 nucleocapsid (red), and NEUN (gray) and counterstained with Hoechst 33342. Scale bar, 10 μm. Yellow arrows indicate infected neurons. (**K**) 3D-reconstruction SARS-CoV-2–positive syncytia with maximum *Z*-plane projection. White numbers mark individual nucleus. Scale bar, 10 μm. (**L**) Graph showing the percentage of SARS-CoV-2–positive syncytia after 72 hpi. Data are means ± SEM. *P < 0.05 via Student’s *t* test. Fifteen ChPCO sections from *n* = 3.

Given that DS individuals are more susceptible to SARS-CoV-2 (*24*, *25*), we next wished to assess the utility of ChPCOs for modeling of SARS-CoV-2 infection of the human CNS. We first interrogated our RNA-seq datasets of uninfected euploid and DS ChPCOs for expression of the host genes that determine SARS-CoV-2 cell entry. SARS-CoV-2 can penetrate susceptible cells via the endosomal entry pathway, which requires the virus binding receptor ACE2, and is facilitated by cleavage of viral Spike protein by the protease furin. Alternatively, the virus can enter the cells via the cell surface pathway (direct fusion), which additionally requires cleavage of the spike protein by TMPRSS2 (*52*). Our RNA-seq data revealed robust expression of the *ACE2* gene in DS organoids at a level similar to that observed in euploid organoids (Fig. 8A). As expected, for a gene located on HSA21, *TMPRSS2* expression was significantly higher in DS than in euploid organoids (Fig. 8A). We further found that *FURIN* expression in DS organoids was threefold increased as compared to the euploid organoids (Fig. 5A), although *FURIN* gene is not located on HSA21. Western blot analysis corroborated the higher levels of TMPRSS2 and FURIN abundance in DS organoids as compared to euploid organoids (Fig. 8B). Immunostaining similarly confirmed much higher expression levels of ACE2, TMPRSS2, and FURIN in ChP-like epithelium than in the cortical tissue portion of the ChPCO organoids (Fig. 8C and fig. S6G). These data therefore indicated that our ChPCOs express receptors and proteases required for SARS-CoV-2 infection, and thus would likely constitute a suitable experimental model for studying SARS-CoV-2 CNS infection in individuals with DS. It also suggests that ChP-like epithelium may facilitate virus entry into CNS as it expressed higher levels of *TMPRSS2* and *FURIN* than neurons.

To assess the effect of ChP-like epithelium on susceptibility of brain organoids to SARS-CoV-2 infection, we infected day 28 euploid and DS ChPCOs with 10^6^ focus-forming units (FFUs) of SARS-CoV-2 for 72 hours (Fig. 8D and fig. S9D) and quantified the expression of SARS-CoV-2 *nucleocapsid*, *RdRp* (Fig. 8E), *Envelope*, and *Spike* genes (fig. S6H) via quantitative reverse transcription PCR (RT-PCR). This revealed that COs were poorly infected by SARS-CoV-2 as compared to ChPCOs and that DS ChPCOs showed significantly higher expression of viral genes than euploid ChPCOs.

To confirm productive SARS-CoV-2 infection, we examined viral titers in the culture supernatants at 0, 24, 48, and 72 hours post-infection (hpi), revealing a significant increase in titers of infectious virus in DS than in the euploid group at 24 and 48 hpi (Fig. 8F). Next, we examined the spatial distribution of SARS-CoV-2–infected cells in ChPCOs. Upon SARS-CoV-2 infection, DS organoids showed a significantly higher proportion of cells expressing SARS-CoV-2 spike protein than euploid organoids (Fig. 8, G and H), consistent with higher viral RNA levels (Fig. 8E and fig. S6H). Immunostaining of EU79- and DS18-derived COs further confirmed the presence of few cortical cells infected with SARS-CoV-2 (fig. S9C). This is consistent with previous studies showing higher SARS-CoV-2 infectivity in organoids with the ChP-like tissue compared to brain organoids (*10*, *11*). We further quantified the percentage of ChP-like epithelia and cortical tissue in ChPCOs during the SARS-CoV-2 experiments. Similar to the above data (Fig. 6C), we found similar cell proportions across groups (fig. S8, C and D), with insignificant increase in ChP-like epithelium in EU79 organoids infected with SARS-CoV-2. These findings indicate that the increased SARS-CoV-2 infection in DS ChPCOs is not attributable to an increase in ChP cells. Given that ChPCOs comprise both ChP-like epithelium and cortical tissues, and since SARS-CoV-2 relevant receptor and proteases are more highly expressed in ChP-like epithelium than in cortical tissues (Fig. 8C and fig. S6G), we next compared the cellular infection vulnerability to SARS-CoV-2 in ChP-like epithelium versus cortical tissue within ChPCOs. Consistently, the number of SARS-CoV-2 spike protein–positive cells in DS ChP-like epithelium and cortical tissues was significantly higher than those in euploid organoids (Fig. 8I). We further stained these organoids with a pan-neuronal marker (NEUN) and found that many cortical neurons in the cortical tissue had been infected with SARS-CoV-2 (Fig. 8J, yellow arrows). We observed that many of these infected neurons exhibited fragmented nuclei (fig. S7, D and E), suggesting that infection with SARS-CoV-2 induces neuronal cell death.

Next, we examined the cellular consequences of SARS-CoV-2 infection of ChPCOs. We observed the presence of syncytia in SARS-CoV-2–infected cells (Fig. 8K and fig. S6I), which was significantly increased in DS compared to the euploid ChPCOs (Fig. 8L). It is interesting to note that all syncytia were observed in the cortical tissue–infected cells of DS ChPCOs (Fig. 8, K and L, and fig. S6I), while no syncytia were evident in the ChP-like epithelium. By examining individual confocal *Z* planes, we could identify as many as eight nuclei within single infected DS cortical cells at 72 hpi (Fig. 8K). Collectively, our data show that by using ChPCOs as a 3D human cellular model, we were able to reveal significant SARS-CoV-2 tropism for ChP-like epithelium cells, which results in productive infection of cortical tissue cells and increased syncytia in the DS group that may promote viral spread through cell-cell fusion in the brain.

### Transcriptional dysregulation of DS ChPCOs upon SARS-CoV-2 infection

**Fig. 9.**
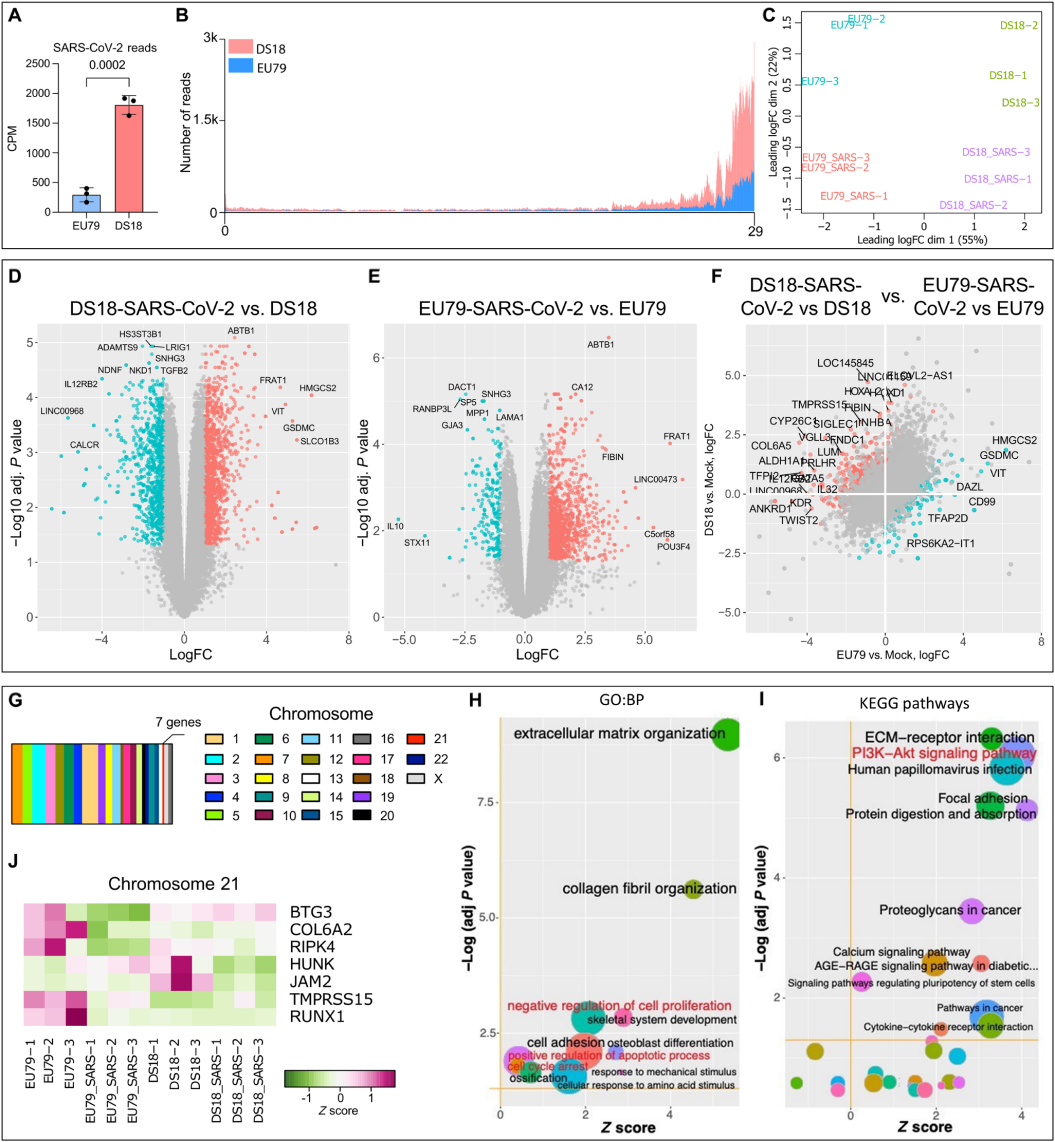
Transcriptional analysis in euploid and trisomy 21 ChPCOs upon SARS-CoV-2 infection. (**A**) Abundance of SARS-CoV-2–derived RNA-seq reads in euploid (EU79) and DS (DS18) ChPCOs. Read counts were normalized to the library sizes. Error bars indicate SD. Statistical analysis was performed by Student’s *t* test. (**B**) Distribution of SARS-CoV-2–derived RNA-seq reads across viral genome. Data for DS (DS18) ChPCOs are shown in red, and euploid (EU79) ChPCOs are in blue. (**C**) Distribution multidimensional scaling analysis of RNA-seq read counts derived from uninfected and SARS-CoV-2–infected human ChPCOs of euploid (EU79) and DS (DS18). (**D** and **E**) Volcano plots highlight DEGs in SARS-CoV-2–infected DS (D) and euploid (E) ChPCOs compared to mock. (**F**) Scatterplot indicating the differences in SARS-CoV-2–induced gene expression changes between DS18 and EU79 ChPCOs. In (D) to (F), the significantly (FDR-adjusted *P* < 0.05) up- and down-regulated genes with at least twofold change in expression levels are shown in red and cyan, respectively. Most DEGs are labeled. (**G**) Distribution of the SARS-CoV-2–responsive DEGs identified in (F) across individual chromosomes. (**H**) GO and (**I**) KEGG pathway enrichment analysis of DEGs in (F). *Z* scores indicate the cumulative increase or decrease in expression of the genes associated with each term. Size of the bubbles is proportional to the number of DEGs associated with respective GO term. (**J**) Expression of the chromosome 21–associated genes identified in (F) in SARS-CoV-2–infected and uninfected DS18 and euploid (EU79) ChPCOs.

To gain additional insight into the cellular responses to SARS-CoV-2 infection in ChPCOs, we performed RNA-seq of day 31 euploid and trisomy 21 ChPCOs that were exposed to SARS-CoV-2 for 72 hours. We first examined the reads that map to the SARS-CoV-2 genome and found a significantly higher load of SARS-CoV-2 RNA in DS ChPCOs as compared to the euploid organoids (Fig. 9A). The majority of virus-derived reads mapped to the 3′ terminal part of the viral genome, which represents a subset of actively transcribed subgenomic RNAs, thus further confirming viral RNA replication in the infected cells (Fig. 9B). Principal components analysis showed good clustering of biological replicates within different groups and separation of samples based on genotype in the first dimension and SARS-CoV-2 infection along the second dimension (Fig. 9C). This analysis further revealed that the euploid and trisomy 21 SARS-CoV-2–positive organoids were transcriptionally different from uninfected organoids in both groups (Fig. 9C). Hierarchical clustering analysis using Spearman rank correlation of the top 500 most variable genes demonstrated that the SARS-CoV-2–positive organoids in the euploid and DS groups were transcriptionally different from the other two SARS-CoV-2–negative groups (fig. S7A). Compared to the euploid organoids with SARS-CoV-2, we also confirmed the significant up-regulation of *TMPRSS2* and *FURIN* (fig. S7B), while *ACE2* remained at a comparable expression level between the two groups (fig. S7B). Comparison of SARS-CoV-2–infected and control uninfected DS ChPCOs revealed 1058 up-regulated genes and 215 down-regulated genes (Fig. 9D and table S2). Comparison of SARS-CoV-2–infected and control uninfected euploid ChPCOs revealed 1118 up-regulated genes and 758 down-regulated genes (Fig. 9E and table S3), indicating that SARS-CoV-2 infection leads to large-scale transcriptional dysregulation.

We next investigated unique SARS-CoV-2–responsive genes that act in DS but not in euploid organoids. We identified 392 up-regulated genes and 219 down-regulated genes (Fig. 9F and table S4). Notably, these genes were distributed across the entire genome, with only seven of these located on chromosome 21 (Fig. 9, G and J). The gene ontology and pathway enrichment analyses of the identified DEGs revealed that DS18 ChPCOs exhibit substantially stronger activation of the processes related to negative regulation of cell proliferation, positive regulation of apoptotic process, cell cycle arrest, and phosphatidylinositol 3-kinase (PI3K)–Akt signaling pathways (Fig. 9, H and I, and fig. S7C), consistent with previous data showing that SARS-CoV-2 increases cell death in ChP organoids (*10*). Notably, no activation of IFN pathway was evident in SARS-CoV-2–infected organoids and no difference in expression of IFN-stimulated genes (ISGs) was observed (Fig. 9H and table S4).

Considering the activation of proapoptotic pathways in the infected organoids (Fig. 9H and table S4), the rapid decline in SARS-CoV-2 viral titers at 72 hpi (Fig. 8F) is most likely explained by rapid elimination of permissive cells through programmed cells death rather than by virus clearance via innate immune mechanisms. To address this, we further examined the expression of proapoptotic genes (table S5) and assessed the rate of cell death before and after SARS-CoV-2 infection. Before SARS-CoV-2 infection, DS organoids showed low expression of genes associated with apoptotic processes (fig. S8A), consistent with previous report suggesting that DS cells are less sensitive to apoptosis (*53*). However, exposure of DS organoids to SARS-CoV-2 significantly altered the expression of the proapoptotic genes (fig. S8A). Immunostaining further demonstrated a significant increase in cleaved caspase-3–positive cells following SARS-CoV-2 infection in DS organoids as compared to euploid organoids (fig. S8B). Up-regulation of many genes associated with cell adhesion and ECM remodeling (Fig. 9, F and H) suggests that infected ChP-like epithelial cells in ChPCOs could signal the CSF barrier to promote SARS-CoV-2 invasion (*54*, *55*). Collectively, these transcriptome analyses are consistent with the notion that SARS-CoV-2 productively infects DS ChPCOs and leads to SARS-CoV-2 invasion, defects in cell cycle and proliferation, and increased cell death of cortical neurons.

### TMPRSS2 inhibitors reduce SARS-CoV-2 infection of DS organoids

**Fig. 10.**
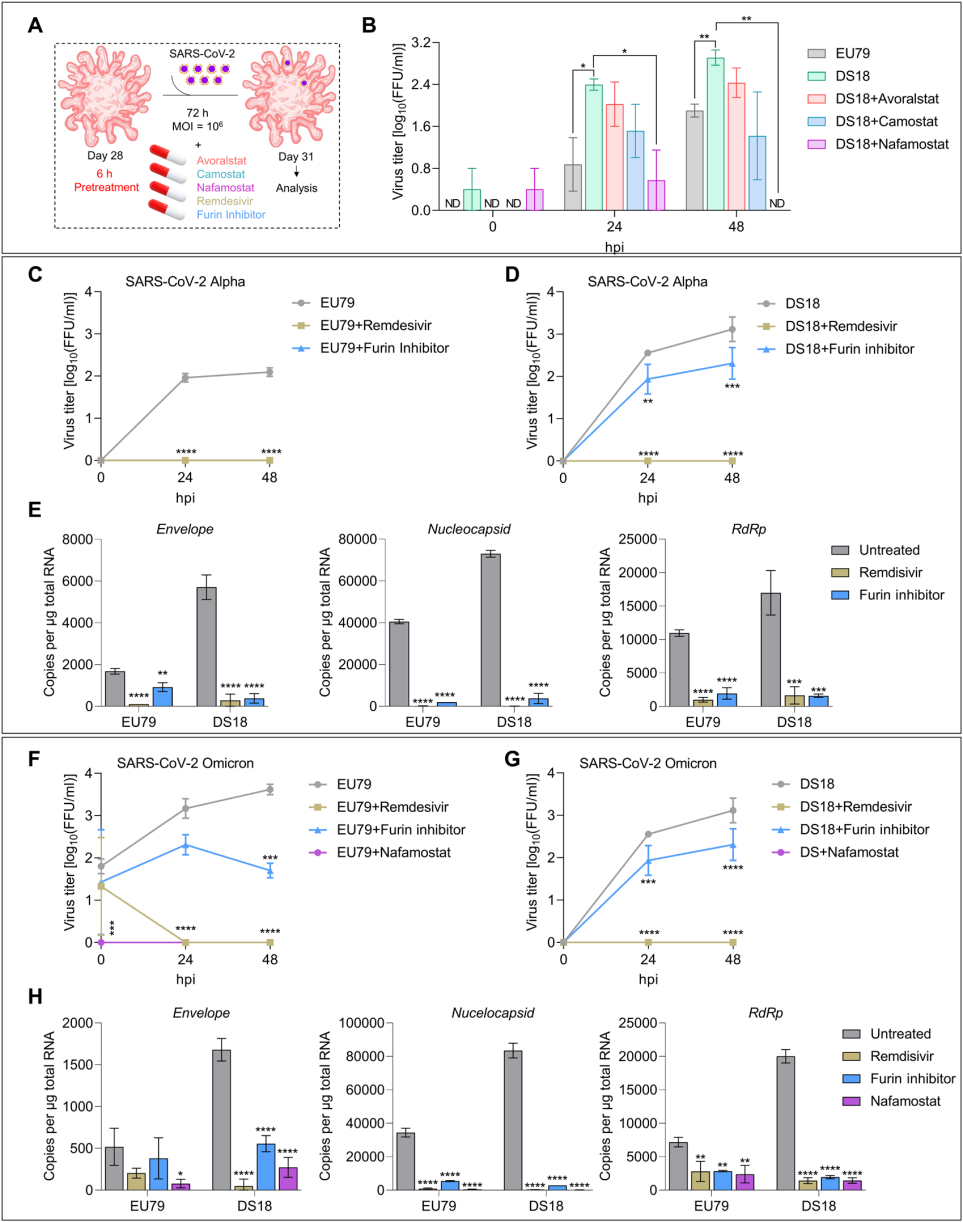
Drug screening of FDA-approved inhibitors against TMPRSS2 activity in ChPCOs. (**A**) Diagram of the SARS-CoV-2 infection protocol. Remdesivir was added immediately after infection and for 72 hours during infection. (**B**) Quantification of viral titers from infected ChPCO supernatants treated with 100 μM TMPRSS2 inhibitors. Data are means ± SEM. *P < 0.05, **P < 0.01 via Student’s *t* test. *N* = 20; organoid number = 60 summarized in table S8. (**C**) Quantification of viral titers from infected ChPCO supernatants treated with 50 μM furin inhibitor and 100 μM remdesivir. Data are means ± SEM. *N* = 3 summarized in table S8; ****P < 0.0001 via two-way ANOVA. (**D**) Quantification of viral titers from infected ChPCO supernatants treated with 50 μM furin inhibitor and 100 μM remdesivir. Data are means ± SEM. *N* = 3 summarized in table S8; **P < 0.01, ***P < 0.0001, ****P < 0.0001 via two-way ANOVA. (**E**) Graphs showing the SARS-CoV-2 *Envelop*, *nucleocapsid*, and *RdRp* in ChPCOs on day 31. Data are means ± SD. **P < 0.01, ****P < 0.0001 via one-way ANOVA. *N* = 3 summarized in table S8. (**F**) Quantification of viral titers from infected ChPCO supernatants treated with 50 μM furin inhibitor, 100 μM remdesivir, and 100 μM nafamostat. Data are presented as means ± SEM. *N* = 3 summarized in table S8. ***P < 0.0001, ****P < 0.0001 via two-way ANOVA. (**G**) Quantification of viral titers from ChPCO supernatants treated with 50 μM furin inhibitor, 100 μM remdesivir, and 100 μM nafamostat after SARS-CoV-2 Omicron (10^6^ FFUs) infection. Data are means ± SEM. *N* = 3 summarized in table S8. ***P < 0.001, ****P < 0.0001 via two-way ANOVA. (**H**) Graphs showing the SARS-CoV-2 Omicron genes *Envelop*, *nucleocapsid*, and *RdRp* in EU79 and DS18 ChPCOs on day 31. Data are means ± SD. *P < 0.05, **P < 0.01, ****P < 0.0001 via one-way ANOVA. *N* = 3 summarized in table S8.

The transcriptional dysregulation in DS due to the triplication of HSA21 likely results in higher risk for more severe COVID-19, which may at least in part be due to the increased production of TMPRSS2 (*31*). Since we detected a threefold increase of TMPRSS2 in DS organoids (Fig. 8, A and B), we next evaluated the effects of the TMPRSS2 inhibitors avoralstat, camostat, and nafamostat on productive SARS-CoV-2 infection (Fig. 10A). DS ChPCOs were pretreated with inhibitors for 6 hours at an effective dose, and treatment continued for the next 72 hours during SARS-CoV-2 infection, as suggested in previous studies (*56*, *57*). We next compared the viral titers at 24 and 48 hpi to those produced in SARS-CoV-2–infected euploid and DS18 organoids not treated with inhibitors. All inhibitors significantly reduced viral titers in the supernatants of DS ChPCOs at 24 and 48 hpi to a level comparable to that in SARS-CoV-2–infected euploid organoids (Fig. 10B). Nafamostat exhibited the strongest inhibition of SARS-CoV-2 infection in DS organoids compared to avoralstat or camostat (Fig. 10B), and this resulted in complete elimination of infectious virus from organoids at 48 hpi (Fig. 10B). Notably, treatment with any of the three TMPRSS2 inhibitors completely abolished virus replication in euploid organoids (fig. S8E). Collectively, these results indicate that TMPRSS2-mediated virus pathway (direct membrane fusion) is the predominant entry way for SARS-CoV-2 infection in brain tissue. Furthermore, we aimed to test whether nafamostat-pretreated organoids exhibited reduced cell death after SARS-CoV-2 infection. Immunostaining of sectioned organoids identified significant reduction in SARS-CoV-2–positive cells in euploid and DS ChPCOs (fig. S9A). Additionally, we found a significant reduction in cell death, marked by cleaved caspase-3 (fig. S9B). This observation aligns with nafamostat’s function to interfere with the spike protein’s activation, thus hindering viral replication and spread within the body, rather than preventing the initial infection of SARS-CoV-2.

Considering that DS brain organoids also exhibited higher levels of furin compared to euploid control (Fig. 8, A to D), we then assessed the effect of the furin inhibitor decanoyl-RVKR-CMK on viral replication in DS and euploid organoids. Furin cleavage of spike is not a prerequisite for SARS-CoV-2 entry, but it highly facilitates viral infectivity (*58*). Treatment with the furin inhibitor completely abolished replication of SARS-CoV-2 in euploid organoids (Fig. 10, C and E), which indicates that in brain tissue furin activity is essential for SARS-CoV-2 entry via membrane fusion. The same effect was observed after treatment with FDA-approved COVID19 drug remdesivir (Fig. 10, C and E). However, the effect of the furin inhibitor on virus replication in DS organoids was less profound. Although decanoyl-RVKR-CMK caused a significant 10-fold reduction of viral titers at 24 and 48hpi, it did not eliminate the virus completely, while remdesivir did (Fig. 10, D and E). This can be explained by higher availability of TMPRSS2 and furin in DS ChPCOs (Fig. 8, A to C). On the other hand, the evolving SARS-CoV-2 Omicron variants are less efficiently cleaved by furin but exhibit greater sensitivity to TMPRSS2 inhibitor (*59*). This prompted us to assess the effect of the furin inhibitor on Omicron BA.5 replication in DS and euploid organoids. Both nafamostat and remdesivir treatments completely abolished Omicron BA.5 viral replication in DS and euploid organoids (Fig. 10, F to H). Although the furin inhibitor significantly reduced viral replication in both groups (Fig. 10, F and G), the furin inhibitor failed to completely abolish Omicron BA.5 in contrast to nafamostat and remdesivir (Fig. 10, F to H). Collectively, these experiments provide a proof of concept that ChPCOs can be used as a model to screen for drugs that can inhibit SARS-CoV-2 neuropathology in specific genetic backgrounds, in this case for trisomy 21.

## DISCUSSION

Congenital disorders encompass a wide range of ChP pathologies such as ChP cysts, diffuse villous hyperplasia, lipoma, and Sturge-Weber syndrome (*60*). For instance, ChP cysts are common in fetuses with trisomy 18 and Aicardi’s syndrome, where ChP cysts exhibit accumulation of the CSF causing hydrocephalus (*61*). Many of these ChP abnormalities are linked to other structural anomalies in the brain, including cortical development. The development of human in vitro models that enable investigation of the cellular and molecular mechanisms underlying such diseases, particularly those in which the early stages of ChP development and function are impaired, is therefore valuable and may allow the development of therapeutic strategies that mitigate disruption of the ChP function during development or later in life. Here, we developed a rapid and robust protocol that generates organoids with multiple ChP-like epithelia, which form ventricles that enclose developing functional cortical neuronal cells. We show that the ChP-like epithelial layer that surrounds these organoids exhibits the typical features of the mature in vivo ChP both at the cellular and molecular levels. The cortical cells (progenitors and neurons) and ChP-like epithelial cells of these ChPCOs arise from a common progenitor, the hNEct cells, that can be readily expanded and genetically manipulated with CRISPR to create various disease models.

Organoids representing multiple brain domains have been established (*62*, *63*), including ChP (*9*, *10*). Pellegrini *et al*. (*9*) showed the presence of cortical neurons in their ChP organoids using single-cell RNA-seq, and ChP organoids were subsequently used to demonstrate the susceptibility of ChP-like epithelium to SARS-CoV-2 pathology (*10*, *11*). BMP4 signaling is known to instruct neuroepithelia to become ChP (*36*) at the expense of neural lineages (*9*, *10*). Recent studies used a high concentration of BMPs to generate organoids that almost entirely consisted of ChP-like epithelial cells (*9*, *10*). In contrast, we used an approximately 10-fold lower BMP4 concentration to allow neural lineage specification and further differentiation into cortical neural cells while still permitting ChP-like epithelium development in ChPCOs. Consistent with this notion (fig. S1D), we demonstrated that different concentrations of BMP4 altered the content of neural progenitors. Recently, the in vitro generation of hNEct from hPSCs has been instrumental in the production of forebrain cells in conventional monolayer culture and in 3D organoid models (*35*, *64*, *65*). Here, we took advantage of the developmental potential of hNEct sheets to generate a complex 3D ChPCO model, in which two different brain domains, cortical neurons and ChP-like epithelium, develop in parallel from common hNEct progenitors that self-organize, and interact to promote the formation of ventricle-like structures. Different hPSC lines were used to generate ChPCOs, demonstrating the reproducibility of this protocol. ChPCOs contain key components of the mature ChP in vivo including the establishment of apicobasal polarity. Transcriptomic data of ChPCOs over time suggest the ability of ChPCOs to produce CSF, and our multiplexed ELISA analyses of CST3, APP, and B2M in ChPCO medium confirm secretion of these CSF proteins. We also showed that the evolutionary conserved anti-aging protein KLOTHO is expressed in human ChPs of ChPCOs. Klotho is known to protect against multiple neurological and psychological disorders (*34*). We further demonstrated that human ChP-like epithelial cells in ChPCOs project up to nine cilia per cell during differentiation, with a significant shift from primary to 80% multicilia and a mean length of a cilium increasing from 0.8 to 3 μm over time. This finding aligns with a recent study that reported, over time, that in vivo ChP cells increased the percentage of multiciliated cells to 80% and their length to 3 μm (*66*). Additionally, this study revealed that ChP cilia maintain a 9 + 0 structure throughout life, while axonemal cilia (9 + 2) gradually disappear after weaning. However, our data show no significant changes in genes involved in cilia resorption over time. Given that nodal cilia are typically found in embryonic development and play a crucial role in determining the left-right asymmetry of the body, and axonemal cilia are involved in movement and fluid flow over cell surfaces in various tissues and organs, these data suggest that these ChPCOs are closely associated with fetal human ChP and thus likely have both nodal and axonemal cilia, with no regressing axonemal cilia. Further research is required to ascertain whether these cilia subtypes are of the motile 9 + 2 axonemal, nonmotile 9 + 0 axonemal, or 9 + 0 motile nodal-like variety. ChPCOs described herein should provide a valuable model for exploring axoneme organization and diversity in subsets of human ChP-like epithelial cells. This may shed light on their respective roles in ChP function and cortical development, as well as in the development of human brain defects such as hydrocephalus.

The distribution of SOX2-positive cells in the cortical tissue of ChPCOs is scattered, found both in proximity to and at a distance from the ChP-like epithelium. These SOX2-positive cells contribute to the formation of different cortical neuronal layers during differentiation. Consequently, these cortical neurons do not exhibit a pattern or layering similar to that found in the endogenous brain. This highlights a notable observation regarding the architectural organization of the cortical tissue within these organoids. It became evident that the patterning of the cortical tissue in the organoids does not mirror the complex and precise organization observed in the endogenous cortical tissue of the developing brain (Fig. 4I). The data suggest that while the organoids successfully mimic certain aspects of the brain’s structure, this limitation highlights a critical area for further research that could potentially guide improvements in organoid technology. The presence of developing functional cortical cells that are juxtaposed to ChP-like epithelial cells in ChPCOs has the distinct advantage that it allows investigation of ChP-mediated neurodevelopmental defects such as ventriculomegaly in DS and hydrocephalus in Bardet-Biedl syndrome with patient-specific iPSC lines or genome-edited control iPSC. Because such neurodevelopmental defects can occur very early during embryo development, they have been difficult to study in a human setting and our model offers unique opportunities to uncover novel disease processes and to assess the effectiveness of pharmacological interventions designed to rescue ChP malformations and promote normal cortical brain development. The future challenge will be to control anterior-posterior identity of hNEct-derived ChP-like epithelium while maintaining maturation of cortical neurons. The ability to fuse different domains of the brain using the organoids system has been recently achieved and was used to analyze complex neurodevelopmental defects (*67*, *68*). Similarly, the establishment of ChPCOs with an LV should enable the establishment of the 4VChPs with hippocampus, for example, through judicious sonic hedgehog activation, and may offer an opportunity to establish and study the anterior-posterior identity with complex networks in an all-brain in vitro 3D model in the future.

We successfully produced ChPCOs using various hPSC lines and an iPSC line, demonstrating the protocol’s robustness in inducing ChP across different experimental settings (fig. S2C). Moreover, these ChPCOs can be cultured and further matured for extended periods, currently up to 1 year. ChPCOs encompass essential and functional elements of forebrain tissue (Figs. 4 and 5). These characteristics make ChPCOs an appealing model system for investigating the role of ChP in neurodevelopmental abnormalities and neuro-inflammatory processes.

In the developing forebrain, Olig2 is expressed ventrally in neural stem cells, and later in development is expressed dorsally to give rise to oligodendroglial cells (*51*). OLIG2 is an HSA21 gene that was recently shown to be overexpressed in neural stem cell populations of ventral forebrain organoids derived from DS iPSCs (*21*). In contrast, we rather found down-regulation of OLIG2 in ChPCOs derived from DS iPSCs at both the gene and protein levels as compared to euploid organoids. It is not unlikely that this difference is due to the dorsal forebrain identity of ChPCOs generated in this study, and we hypothesize that the down-regulation of OLIG2 in DS ChPCOs may underlie down-regulation of OPC markers (*SOX10* and *PDGFRA*) in this model. We further discovered defective cilia formation and disrupted ChP-like epithelial polarity in ChPCOs derived from DS lines. Genes associated with CSF production were found to remain unaltered between DS and euploid organoids, consistent with a previous study (*69*). Therefore, the defective polarity in the ChP-like epithelium in DS organoids might contribute to the changes observed in ion transport–associated genes within DS organoids. We found that ChPCOs derived from different DS iPSC lines exhibited different degree of penetrance of DS pathology. This is consistent with previous studies indicating variable disease penetrance in individuals with DS (*70*) and further supports the notion that the severity of DS phenotypes is affected by genetic background. DS fetuses and 2D neuronal cultures and cortical brain organoids derived from DS hiPSC exhibit an increased production of astrocytes (*16*, *71*). In contrast, analysis of our RNA-seq data of top 500 most variable genes did not detect changes in the expression level of genes associated with astrocytes (table S1). We speculate that this inability to phenocopy this aspect of DS brain development with ChPCOs may be due to the fact that the ChPCO protocol involves prolonged exposure to BMP4 (Fig. 1A), which is known to promote astrocyte differentiation (*72*) and therefore may have obscured intrinsic differences in astrocyte production between DS and euploid groups. We observed up-regulation in cell adhesion and ECM remodeling seen in DS ChPCOs, evident both with (Fig. 9, F and H, and fig. S6K) and without (Fig. 6E) SARS-CoV-2 infection. This aligns with previous studies that report extensive dysregulation of molecules involved in cell adhesion and ECM organization in DS cells such as cardiac cells (*73*), skin fibroblasts (*74*), and umbilical cord (*75*), which is thought to offer protection against certain types of cancers in individuals with DS (*76*). In the brain, DS neural cells, including astrocytes (*77*) and neurons (*78*, *79*), exhibit dysregulation in genes related to cell adhesion and ECM, while neural progenitors (*77*) do not. This suggests that changes in cell adhesion and ECM remodeling may occur globally in the DS brain, potentially influencing brain development and function in individuals with DS.

Viral infection acquired antenatally can have devastating impacts on the developing fetal brain (*80*). Several clinical reports of infants born to women with SARS-CoV-2 infection during pregnancy displayed developmental delay in 10% of infants at 12 months of age (*81*, *82*). Notably, the neurodevelopmental morbidity was not associated with prematurity, suggesting a specific mechanisms of SARS-CoV-2 neurotropism rather than simply contributing to pregnancy complications (*83*). In vitro, SARS-CoV-2 was shown to have neuroinvasive and neurotropic attributes for the human ChP (*10*), neurons (*84*), and astrocytes (*85*), indicating the potential of SARS-CoV-2 to affect early brain development. Given the fact that *TMPRSS2* is located on chromosome 21, and that epidemiological studies have found a distinct vulnerability of people with DS toward SARS-CoV-2, as illustrated by the 4-fold increased risk for COVID-19–related hospitalization and 10-fold increased risk of death (*24*, *25*), we used our DS ChPCOs as a model to study SARS-CoV-2 infection. Upon exposure to SARS-CoV-2, we observed robust infection of the ChP-like epithelium in ChPCO-derived from DS iPSCs and consequent invasion into the cortical tissue. Since the COs were poorly infected, it is likely that ChP-like epithelial cells in ChPCOs serve as viral “replication hubs” that support viral invasion and spread to other cortical cells, consistent with a previous study indicating that COVID-19 neurological symptoms in postmortem brains are linked to perturbations in barrier cells of ChP (*86*). We confirmed that in this model TMPRSS2 and FURIN, but not ACE2 and NRP1, are significantly up-regulated in ChP-like epithelium of DS ChPCOs. As discussed above, DS ChPCOs also display defective epithelial polarity of ChP. The tight junctions in ChP epithelial polarity serve as a barrier to invaders and hamper virus endocytosis. Therefore, both increased dosage of TMPRSS2 as well as defective tight junctions in ChP-like epithelium of DS organoids may combine to promote SARS-CoV-2 entry and subsequent neuropathology. Gene expression studies demonstrated genome-wide transcriptome deregulation in DS individuals, DS iPSCs, and the DS Ts65D mouse model (*87*). Individual with DS develop severe complications such as excessive immune response during viral respiratory infections (*29*). It was recently demonstrated that type I IFN (IFN-I) expression in ChP is age dependent and negatively affects brain function (*88*). IFN-I expression profile was also found in human ChP (*88*). These data identified chronic aging-induced IFN-I signature at ChP, which is often associated with antiviral response (*88*). On the other hand, individuals with DS develop severe complications during viral infection due to impaired immune response (*53*). In the absence of any detectable infections, DS individuals exhibit chronic hyperactive IFN response (*89*), and elevation of many cytokine and chemokine levels known to act downstream of IFN signaling (*90*). This is in a part due to the fact that IFN receptors (IFNRs) are encoded on HSA21 (*89*). While we found that several HSA21 genes (MX2, TMPRSS2, ADAMTS5, and RUNX1) associated with viral infection are expressed in ChPCOs, we failed to detect deregulation of IFN receptors and IFN signaling pathway molecules, and a general lack of expression of IFN-associated genes in our 31-day-old ChPCO and in SARS-CoV-2–infected ChPCOs. Instead, we observed activation of apoptotic pathways and death of infected ChPCO cells (fig. S8, A and B). However, it is important to note that ChP exhibits a widespread up-regulation of inflammatory genes across various IFN-related pathways upon SARS-CoV-2 infection (*8*). Therefore, using spatial transcriptomics or separating the cortical and ChP-like sections from day 31 ChPCOs might reveal whether specific sets of inflammatory genes are enriched solely in the DS ChP-like components.

We further showed that inhibition of TMPRSS2 using FDA-approved drugs (avoralstat, camostat, nafamostat) strongly decreased SARS-CoV-2 replication in all organoids, indicating that TMPRSS2 is required for viral entry into the cells in EU and DS ChPCOs. Consistent with previous reports (*91*), we found a higher potency of nafamostat for inhibiting SARS-CoV-2 infection and replication as compared to avoralstat and camostat. Human neurons have been previously reported to express low levels of ACE2 and no TMPRSS2 (*92*). Here, we found that ChP-like epithelium exhibits high expression of TMPRSS2 and furin, which further increases in DS (Fig. 8, A and B). Our data show that TMPRSS2 inhibition strongly affects virus infection and replication, further underlining the substantial role of this receptor for SARS-CoV-2 infection of ChPCOs and providing additional evidence that TMPRSS2-expressing ChP-like epithelial cells act a primary reservoir for SARS-CoV-2 infection and spread. Inhibition of furin also substantially reduced viral replication in DS ChPCO and, in euploid ChPCO, completely eliminated the virus. Given that cleavage of viral spike protein by furin is known to facilitate viral entry, while not being absolutely essential, these data indicate that DS ChPCO likely have sufficient amount of TMPRSS2 to enable viral entry in absence of the furin activity; however, in euploid organoids that have less TMPRSS2, furin may, however, be strictly required for SARS-CoV-2 infectivity. In addition, we showed that treatment with FDA-approved COVID-19 drug remdesivir results in complete elimination of the virus from both euploid and DS ChPCOS. This is consistent with a high efficacy of the drug against SARS-CoV-2 (*93*).

Our findings reveal an elevated susceptibility to SARS-CoV-2 infection in the DS ChPCOs, consistent with clinical studies reporting medical vulnerability of individuals with DS to severe COVID-19 (*25*, *94*). This heightened susceptibility to SARS-CoV-2 infection in individuals with DS may be intricately tied to their established health vulnerabilities, for instance, individuals with DS often exhibit compromised immune systems that can an impede the body’s ability to mount a robust defense against the virus, potentially allowing its entry into the brain, as seen in this study. In addition, the increased susceptibility of individuals with DS to SARS-CoV-2 can be compounded by the presence of heart defects and respiratory issues, which are common preexisting conditions in this population. For instance, DS individuals with heart defects may experience compromised cardiovascular function, leading to decreased oxygen supply to vital organs; SARS-CoV-2 primarily affects the respiratory system, a system that is limited in function with structural abnormalities in DS; and compromised cardiovascular function may exacerbate the strain on the heart, potentially leading to more severe outcomes during SARS-CoV-2 infection. The interplay of these factors highlights the intricate relationship between preexisting health conditions and the heightened susceptibility to severe outcomes upon SARS-CoV-2 infection in individuals with DS. These health challenges may collectively amplify the impact of SARS-CoV-2 on the neurological system, emphasizing the importance of considering the broader health context when assessing the risks and implications of viral infections in this vulnerable population.

In conclusion, our study underscores the relevance of ChPCOs for modeling neurodevelopmental diseases such as DS and (corona)virus research, and signifies the importance of protease proteins (TMPRSS2, FURIN) as attractive therapeutic targets for inhibiting SARS-CoV-2 neurotropism. ChPCOs should further prove useful for identifying and screening therapeutics for future emerging viruses and for modeling congenital disorders that involve ChP dysfunction.

## MATERIALS AND METHODS

### Human embryonic stem cell culture and CO generation

hESC H9 (from Wisconsin International Stem Cell Bank, WiCell Research Institute, WA09 cells), WTC iPSC (gift from B. Conklin), G22, DS18, and EU79 iPSC lines (available in our laboratory) were cultured according to STEMCELL Technologies protocols (which can be found at https://www.stemcell.com/maintenance-of-human-pluripotent-stem-cells-in-mtesr-1.html) on feeder-free hESC medium on Matrigel (STEMCELL Technologies, catalog no. 354277) in mTeSR (STEMCELL Technologies, catalog no. 85851), as we reported recently (*95*).

### ChP-CO generation

To generate ChPCOs, hPSC colonies were plated on a hESC qualified basement membrane matrix (STEMCELL Technologies, catalog no. 354277) in a six-well plate at 20% to 30% density (*96*). hPSC colonies were maintained with mTeSR for 1 day before NEct induction. To generate NEct colonies, hPSC colonies were cultured for 3 days in N2 medium: Dulbecco’s modified Eagle’s medium (DMEM)/F12 (Gibco, catalog no. 11320-33), 2% B-27 supplement (Gibco, catalog no. 17504044), 1% N-2 supplement (Gibco, catalog no. 17502-048), 1% MEM nonessential amino acids (Gibco, catalog no. 11140-050), 1% penicillin/streptomycin (Gibco, catalog no. 15140148), and 0.1% β-mercaptoethanol (Gibco, catalog no. 21985-023), containing dual SMAD inhibitors, SB-431542 (10 μM), and LDN 193189 (100 nM). Fresh N2 medium with inhibitors was added daily. On the fourth day, induced NEct colonies were lifted using dispase (2.4 U/ml) to form neural spheroids as we recently reported (*35*). For the next 4 days, these spheroids were cultured in N2 medium supplemented daily with bFGF (40 ng/ml; R&D Systems, catalog no. 233-FB-01M), 2 μM CHIR99021 (Sigma-Aldrich, catalog no. SML1046-5MG), and BMP4 (2 ng/ml) (Thermo Fisher Scientific, catalog no. PHC9391). Patterned neural spheroids were then embedded in Matrigel (STEMCELL Technologies, catalog no. 354277) and switched to the terminal differentiation medium DMEM-F12 (Gibco, catalog no. 11320-33): Neurobasal medium (Gibco, catalog no. A35829-01), 0.5% N2 (Gibco, catalog no. 17502-048), 12.5 μl of insulin in 50-ml medium (Sigma-Aldrich), 1% GlutaMAX, 1% MEM nonessential amino acids (Gibco, catalog no. 11140-050), 1% penicillin/streptomycin (Gibco, catalog no. 15140148), 17.5 μl of β-mercaptoethanol in 50-ml medium (Gibco, catalog no. 21985-023), and 1% B-27 supplement (Gibco, catalog no. 17504044), supplemented with 3 μM CHIR99021 and BMP4 (5 ng/ml). Fresh medium was replaced three times a week. All experiments were carried out in accordance with the ethical guidelines of the University of Queensland and with the approval by the University of Queensland Human Research Ethics Committee (approval number 2019000159).

### Quantitative RT-PCR

Total RNA was isolated from organoids as described previously (*97*). For qPCR, 1 μg of isolated RNA was used to generate the complementary DNA (cDNA) using the First-Strand cDNA Synthesis Kit (Thermo Fisher Scientific, catalog no. K1612). SYBR Green (Applied Biosystem, catalog no. A25742) was used, and PCR standard reaction conditions were set according to the manufacturer’s instructions. For quantification of viral RNA, 1 μg of total RNA was reverse-transcribed using qScript cDNA SuperMix (Quanta Bio, catalog no. 95548). The cDNA was diluted 1:10, and 3 μl of the solution was used as a template for qRT-PCR. The qPCR was performed using QuantiNova SYBR Green PCR Kit (Qiagen, catalog no. 208056) on Applied Biosystem QuantStudio 6 instrument. RNA copy numbers were determined by comparing sample *C*_t_ values to the standard curve obtained by amplification of the DNA standards generated by end-point RT-PCR with the same sets of primers. PCR primers were designed using the National Center for Biotechnology Information (NCBI) free online system, and all the RT-qPCR primers are listed in table S6. All experiments were performed in biological triplicates for every sample, and the expression values were normalized against the glyceraldehyde-3-phosphate dehydrogenase (GAPDH) expression value of each sample. The means and SDs were calculated and plotted using the GraphPad Prism 9.

### Immunohistochemistry

Tissue processing and immunohistochemistry (IHC) were performed as described in (*98*). In brief, organoids were fixed in 4% paraformaldehyde (PFA) for 60 min at room temperature, followed by washing with 1× phosphate-buffered saline (PBS) three times for 10 min at room temperature. Fixed organoids were then immersed in 30% sucrose in PBS at 4°C and allowed to sink before being embedded in a solution containing a 3:2 ratio of Optimal Cutting Temperature (O.C.T.) and 30% sucrose on dry ice. Mounted tissues were then subjected to serial sections at 14-μm thickness and collected onto Superfrost slides (Thermo Fisher Scientific, catalog no. SF41296). To performed IHC, sectioned organoids were washed three times with 1× PBS for 10 min at room temperature before blocking for 1 hour with 3% bovine serum albumin (BSA) (Sigma-Aldrich, catalog no. A9418-50G) and 0.1% Triton X-100 in 1× PBS. Primary antibodies were added overnight at 4°C before washing three times with PBS for 10 min each at room temperature. For immunocytochemistry (*99*), cells were allowed to be fixed with 4% PFA in 1× PBS for 10 min at room temperature. The cells were then washed three times with 1× PBS at room temperature before blocking and adding primary antibody as stated above. Tissues and cells were then incubated with appropriate secondary antibodies for 1 hour at room temperature before mounting and imaging. The whole mount was performed as described before (*100*). All samples were counterstained with Hoechst 33342 (Invitrogen, catalog no. H3570). All images were acquired using confocal microscopy (Leica TCS SP8) based in School of Biomedical Sciences Imaging Facilities at the University of Queensland. The primary antibodies used in this study are listed in table S7. Alexa Fluor 488–, Alexa Fluor 546–, and Alexa Fluor 633–conjugated secondary antibodies were obtained from Jackson ImmunoResearch Laboratory.

### RNA sequencing

RNA was isolated from the pools of three individual organoids using TRI Reagent (Sigma-Aldrich, USA) as described previously (*101*). RNA integrity was analyzed on TapeStation 4200 (Agilent, USA), and samples with RIN > 8 were used for library preparation with TrueSeq RNA Library Preparation Kit v2, Set A (Illumina, USA). Barcoded cDNA libraries were pooled and sequenced on Illumina NextSeq 500 instrument using NextSeq 500/550 High Output 75 Cycles Kit v2.5 (Illumina, USA). Image acquisition, processing, and fastq demultiplexing were performed using in-instrument software as described previously (*102*).

### Differential gene expression analysis

Quality control of raw sequencing data was performed using FastQC software v.0.72. Reads were then trimmed using Trimmomatic software v.0.36.6 with the following parameters: ILLUMINACLIP : TruSeq3 - SE : 2 : 30 : 10, LEADING : 32 TRAILING : 32, SLIDINGWINDOW : 4 : 20, MINLEN : 16. Trimmed reads were mapped to SARS-CoV-2 genome (GISAID Accession ID: EPI_ISL_944644) using Bowtie2 v.2.4.4. Aligned reads were visualized and quantified using Integrative Genomic Viewer v.2.13.1 (Broad Institute, USA). Human reads were mapped to the genome assembly hg38 using HISAT2 v.2.2.1. Feature counting was performed using featureCounts v2.0.1 with counting mode set to “Union” and strand to “Unstranded,” feature type was “exon,” and ID attribute was Gene_ID.

Differential gene expression analysis was performed using edgeR v.4.2. Low abundance reads [<1 counts per million (cpm)] were removed from the dataset, and data were normalized to library sizes and composition bias using the trimmed mean of M-values (TMM) method. Normalized data were analyzed by multidimensional scaling analysis and used to build quasi-likelihood negative binomial generalized log-linear model. The Treat test (glmTreat) was then applied to the contrasts ChP-NC, DS18_ChP-EU79_ChP, SARS_DS18-Mock_DS18, and SARS_EU79-Mock_EU79 to identify the genes that are differentially expressed between the groups by at least one log_2_. The quasi-likelihood *F* test (glmQLFTest) was applied to the contrast (SARS_DS18-Mock_DS18)-(SARS_EU79-Mock_EU79) to identify the genes affected by infection specifically in DS18 organoids. Genes were considered differentially expressed if false discovery rate (FDR)–corrected *P* values were <0.05. Gene expression data were plotted using ggplot2 v.3.3.2. Gene ontology and Kyoto Encyclopedia of Genes and Genomes (KEGG) pathway enrichment analyses were performed using Database for Annotation, Visualization and Integrated Discovery (DAVID) v6.8. Enrichment data were then combined with expression values, and *z* scores were calculated using the GOplot v.1.0.2 R package and plotted using ggplot2 v.3.3.2. Heat maps were generated using heatmap.2 function of R-package gplots v3.1.2.

### Transmission electron microscopy

TEM was performed according to (*103*). In brief, organoids were in 0.1 M sodium cacodylate buffer in ddH_2_O containing 2.5% glutaraldehyde and 2% paraformaldehyde overnight at 4°C. Organoids were first washed three times in 0.1 M sodium cacodylate buffer for 10 min at room temperature, followed by immersing in 2% osmium tetroxide (2 ml of 4% osmium tetroxide and 2 ml of 0.2 M cacodylate buffer) for 90 min at room temperature. The staining buffer was then replaced with 2.5% potassium ferricyanide (4.8 ml of 0.2 M cacodylate buffer and 4 ml of 6% potassium ferricyanide) for 90 min at room temperature. The organoids were then washed in water three to five times at room temperature until the water is completely clear, before dipping the organoids in the thiocarbohydrazide solution [0.1 g of thiocarbohydrazide (locked cupboard) in 10 ml of water] for 45 min at 40°C. To remove the background stain, organoids were washed four to five times in water at room temperature before immersing the organoids into 2% osmium tetroxide in 0.1 M cacodylate buffer for 30 min at room temperature. Organoids were again washed in water to remove the black background. Washed organoids were then dipped into 1% uranyl acetate overnight at 4°C, followed by incubation in 0.03 M aspartic acid solution for 30 min at 60°C. Incubated organoids were then washed three times for 30 min at room temperature. For embedding, the organoids were dehydrated in a series of ethanol (20%, 50%, 60%, 70%, 80%, 90%, and 100%) for 30 times at room temperature each. Organoids were then infiltrated with Durcupan resin for 12 hours at room temperature. Embedded organoids were then incubated for 48 hours in 60°C before trimming and imaging.

### Multiplexed ELISA

Organoid media for investigations of CSF biomarker (CST3, APP, and B2M) levels were collected on days 7, 14, 21, 28, 42, and 56. The level of CST3, APP, and B2M in collected medium was examined using Human Magnetic Luminex Assays (R&D Systems, catalog no. LXSAHM-03) according to the manufacturer’s instructions and read on a MAGPIX instrument (Luminex Corp.).

### MEA recording and analysis

Whole organoids cultured in terminal differentiation medium were transferred before recording to the MEA plate [4096 channels at cellular resolution (20 μm) over a large area (up to 5.1 × 5.1 mm^2^), with a sampling frequency of 18 kHz per channel, 3Brain] and recorded in a BioCAM X system (3Brain). The medium was removed from each well until only a thin layer remained to allow the attachment of organoids to the electrode grid under the 37°C temperature and 5% CO_2_. During the recording, 50 μM glutamate (Sigma-Aldrich, catalog no. G1251-100G) or 10 μM NMDA (Sigma-Aldrich, catalog no. M3262-25MG) was added to warm medium to stimulate neuron activity in organoids. To record the inhibitory pharmacologic effects on ChPCOs, 50 μM bicuculline (Sigma-Aldrich, catalog no. 14340-25MG) was added to the medium. The baseline activity was recorded without pharmacologic effects. Data were exported as .csv files for analysis using BrainWave Software. For the mean firing rate, the algorithm PTSD (precise timing spike detection) was applied having a threshold eight times the SD above the background noise, with a 2-ms peak lifetime period and 2-ms refractory period imposed after each detected spike. The bursts were detected using 2 ms of maximum spike interval and 5 as a minimum number of spikes.

### Western blot

Western blotting was performed as described previously (*34*). In brief, Pierce RIPA Buffer (Thermo Fisher Scientific, catalog no. 89900) containing a cocktail of protease and phosphatase inhibitors (Roche) was used to lyse the organoids. A sonicator was used to sonicate the organoids, and Pierce bicinchoninic acid (BCA) protein assay kit (Thermo Fisher Scientific, catalog no. 23227) was used to quantify protein concentration according to the manufacturer’s instructions. The extracted proteins were then heated for 10 min at 100°C before loading. Equal protein amount was loaded and separated using Mini-PROTEAN TGX Stain-Free-Gels (Bio-Rad, catalog no. 4568044). iBlot 2 PVDF Mini Stacks (Invitrogen, catalog no. IB24002) was used to transfer the separated proteins. Skim milk (5%) in TBST [20 mM tris-HCl (pH 7.6), 136 mM NaCl, and 0.1% Tween 20] was used to block the membranes for 1 hour at room temperature, before incubation with primary antibodies, which were diluted in 5% BSA for 12 hours at 4°C. The primary antibodies used in this experiment are listed in table S6. Incubated membranes were then washed three times with 1× TBST for 10 min each at room temperature followed by another incubation with secondary antibody diluted 1:5000 in 5% skim milk in 1× TBST for 1 hour at room temperature. Finally, the incubated membranes were washed again three times with 1× TBST for 10 min each at room temperature and visualized with Clarity Western ECL Substrate (Bio-Rad, catalog no. 170-5060).

### SARS-CoV-2 infection of ChPCOs

SARS-CoV-2 isolate QLD1517/2021 (Alpha variant, GISAID accession EPI_ISL_944644), passage 2, was recovered from nasopharyngeal aspirates of an infected individual and provided by the Queensland Health Forensic and Scientific Services, Queensland Department of Health. The obtained isolate was amplified on Vero E6-TMPRSS2 cells to generate virus stock. Virus titers were determined by immunofluorescent focus-forming assay on Vero E6 cells. Organoids were incubated in 500-μl medium containing 10^6^ FFUs of the virus for 6 hours at 37°C, then inoculum was removed, and organoids were washed three times and then maintained in 1 ml of fresh medium. Culture fluids were sampled at 0, 24, 48, and 72 hpi to monitor virus replication. Organoids were preincubated for 6 hours in medium containing 100 μM TMPRSS2 inhibitor (avoralstat, camostat, or nafamostat) or 50 μM furin inhibitor D-RVKR-CMK. After drug treatment, organoids were infected overnight with SARS-CoV-2 at the dose of 10^6^ FFUs per organoid. Remdesivir (100 μM) was added immediately after SARS-CoV-2 infection. Inoculum was then removed, and organoids were washed three times and placed into the medium containing the same concentration of corresponding drug.

### Immunofluorescent focus-forming assay

Tenfold serial dilutions of cell culture fluids were prepared in DMEM supplemented with 2% fetal bovine serum (FBS), and 25 μl of each dilution was used to infect 2 × 10^4^ Vero E6 cells preseeded in 96-well plates. After 1 hour of incubation at 37°C with the inoculum, 175 μl of the overlay medium was added to each well. The overlay medium consisted of 1:1 mixture of M199 medium [supplemented with 5% fetal calf serum, streptomycin (100 μg/ml), penicillin (100 U/ml), and NaHCO_3_ (2.2 g/liter)] and 2% carboxymethylcellulose (Sigma-Aldrich, USA). At 1 day after infection, overlay medium was removed and cells were fixed by submerging in cold 80% acetone (diluted in 1× PBS) for 1 hour at −20°C. The following procedures were handled in PC2. The cell monolayer was dried and blocked for 60 min with Clear Milk Blocking Solution (150 μl per well) (Pierce, USA). Viral spike protein was probed by incubation with CR3022 mouse monoclonal antibody (50 μl per well) diluted 1:1000 for 1 hour, followed by five washes with phosphate-buffered saline containing 0.05% Tween 20 (PBST), 1-hour incubation with 50 μl per well of 1:1000 dilution of goat anti-mouse IRDye 800CW secondary antibody (LI-COR, USA), and another five PBST washes. All antibodies were diluted with Clear Milk blocking buffer (Pierce, USA), and incubations were performed at 37°C. Plates were then scanned using an Odyssey CLx Imaging System (LI-COR) using the following settings: channel = 800 and 700, intensity = auto, resolution = 42 μm, quality = medium, and focus = 3.0 mm. Virus replication foci were then counted, and viral titers were calculated.

### Statistical analysis

Normally distributed data were expressed as means ± SD of independent experiments. For nonnormally distributed data, median ± SD was used to express the values. The number of biological replicates as well as the sample size are indicated in the figure legends. Student’s *t* test and one-way or two-way analysis of variance (ANOVA) were used for comparing two and more than two groups, respectively. The Tukey’s post hoc analysis was applied for comparisons to a single control. The Kolmogorov-Smirnov test was used to assess difference distributions. Statistical analysis was performed using GraphPad Prism 9 software. Minimal statistical significance was defined at *P* < 0.05.
