# Supplementary material for: Choroid plexus defects in Down syndrome brain organoids enhance neurotropism of SARS-CoV-2
Source: Sci Adv. 2024 Jun 5;10(23):eadj4735. doi: 10.1126/sciadv.adj4735 (PMC11152128; doi:10.1126/sciadv.adj4735)
Supplement: Supplementary file 1 — Figs. S1 to S9 Legends for tables S1 to S8 [file sciadv.adj4735_sm.pdf]

Science Advances
AAAS

Supplementary Materials for

Choroid plexus defects in Down syndrome brain organoids enhance neurotropism of SARS-CoV-2

Mohammed R. Shaker *et al.*

Corresponding author: Mohammed R. Shaker, m.shaker@uq.edu.au; Ernst J. Wolvetang, e.wolvetang@uq.edu.au

Sci. Adv. **10**, eadj4735 (2024)
DOI: 10.1126/sciadv.adj4735

The PDF file includes:

Figs. S1 to S9
Legends for tables S1 to S8

Other Supplementary Material for this manuscript includes the following:

Tables S1 to S8

Fig. S1

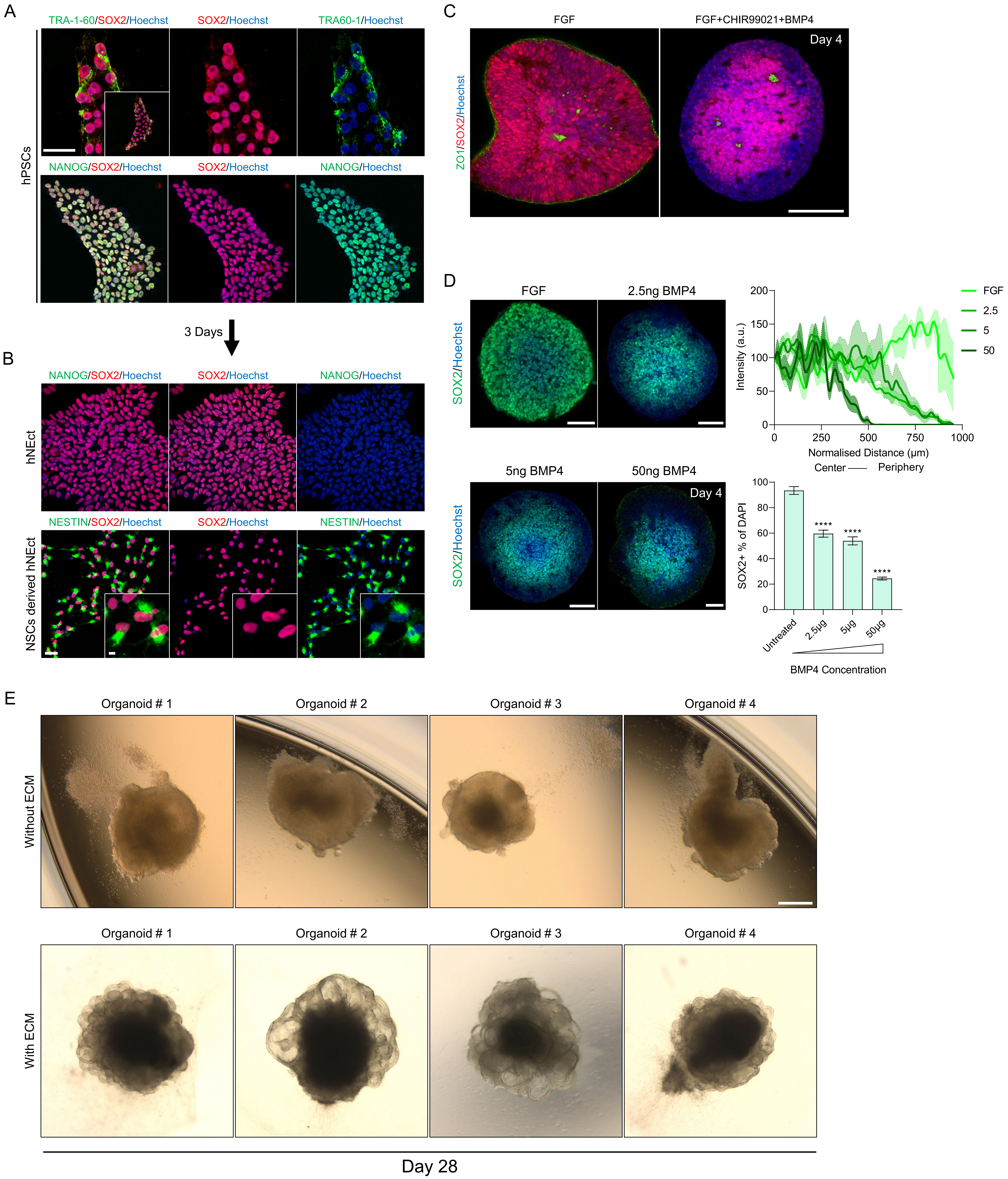

A
TRA-1-60/SOX2/Hoechst
SOX2/Hoechst
TRA60-1/Hoechst
hPSCs
NANOG/SOX2/Hoechst
SOX2/Hoechst
NANOG/Hoechst
3 Days
B
NANOG/SOX2/Hoechst
SOX2/Hoechst
NANOG/Hoechst
hNECt
NESTIN/SOX2/Hoechst
SOX2/Hoechst
NESTIN/Hoechst
NSCs derived hNECt
C
FGF
FGF+CHIR99021+BMP4
Day 4
ZO1/SOX2/Hoechst
D
FGF
2.5ng BMP4
SOX2/Hoechst
Intensity (a.u.)
Normalised Distance (µm)
Center —— Periphery
FGF
2.5
5
50
5ng BMP4
50ng BMP4
Day 4
SOX2/Hoechst
SOX2+ % of DAPI
****
Untreated
2.5µg
5µg
50µg
BMP4 Concentration
E
Organoid # 1
Organoid # 2
Organoid # 3
Organoid # 4
Without ECM
With ECM
Day 28

Fig. S2

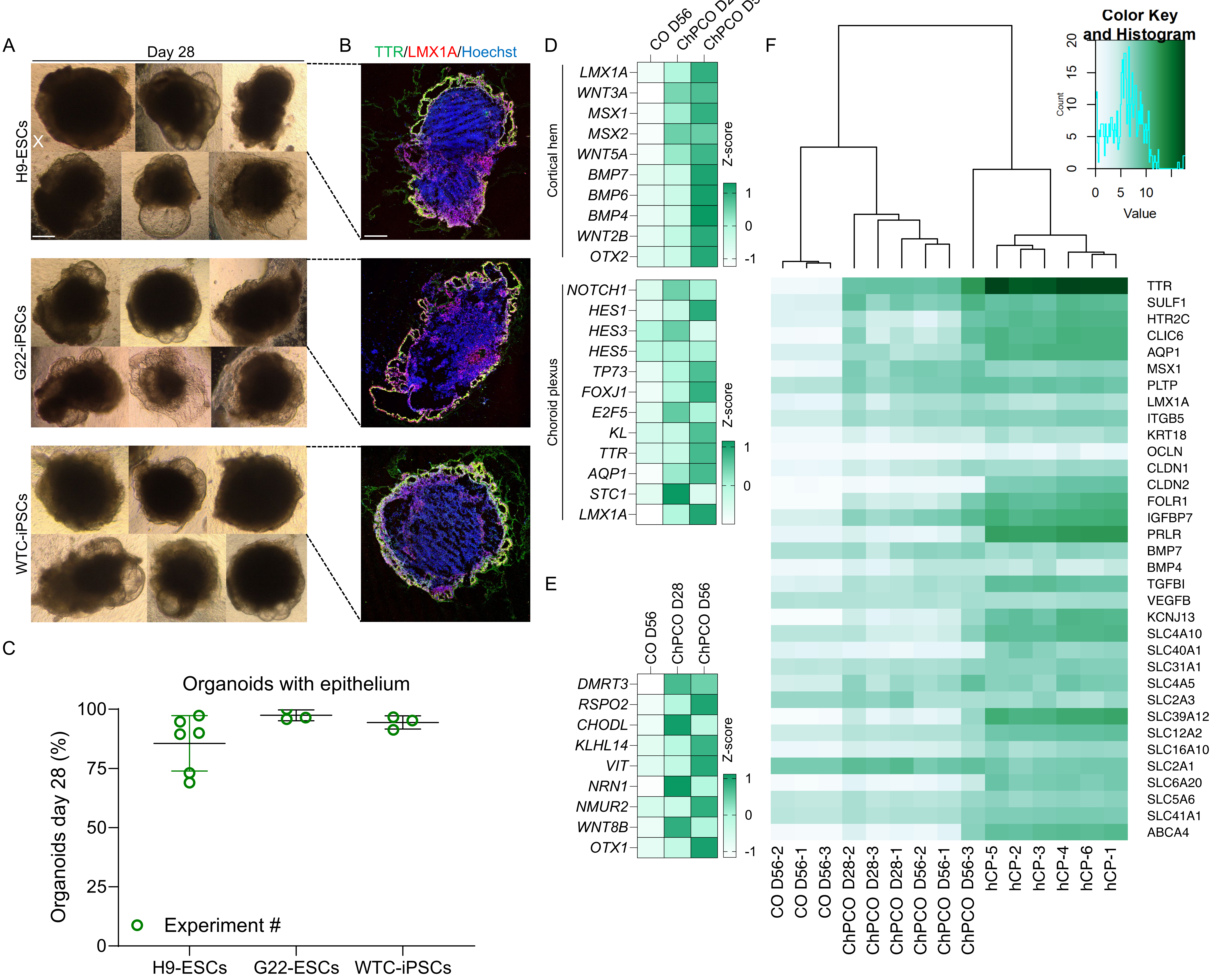
A
Day 28
H9-ESCs
G22-iPSCs
WTC-iPSCs
X
B
TTR/LMX1A/Hoechst
C
Organoids with epithelium
Organoids day 28 (%)
100
75
50
25
0
Experiment #
H9-ESCs
G22-ESCs
WTC-iPSCs
D
CO D56
ChPCO D28
ChPCO D56
Cortical hem
LMX1A
WNT3A
MSX1
MSX2
WNT5A
BMP7
BMP6
BMP4
WNT2B
OTX2
Z-score
1
0
-1
Choroid plexus
NOTCH1
HES1
HES3
HES5
TP73
FOXJ1
E2F5
KL
TTR
AQP1
STC1
LMX1A
Z-score
1
0
E
CO D56
ChPCO D28
ChPCO D56
DMRT3
RSPO2
CHODL
KLHL14
VIT
NRN1
NMUR2
WNT8B
OTX1
Z-score
1
0
-1
F
Color Key and Histogram
Count
0
5
10
15
20
Value
0
5
10
TTR
SULF1
HTR2C
CLIC6
AQP1
MSX1
PLTP
LMX1A
ITGB5
KRT18
OCLN
CLDN1
CLDN2
FOLR1
IGFBP7
PRLR
BMP7
BMP4
TGFBI
VEGFB
KCNJ13
SLC4A10
SLC40A1
SLC31A1
SLC4A5
SLC2A3
SLC39A12
SLC12A2
SLC16A10
SLC2A1
SLC6A20
SLC5A6
SLC41A1
ABCA4
CO D56-2
CO D56-1
CO D56-3
ChPCO D28-2
ChPCO D28-3
ChPCO D28-1
ChPCO D56-2
ChPCO D56-1
ChPCO D56-3
hCP-5
hCP-2
hCP-3
hCP-4
hCP-6
hCP-1

Fig.S3

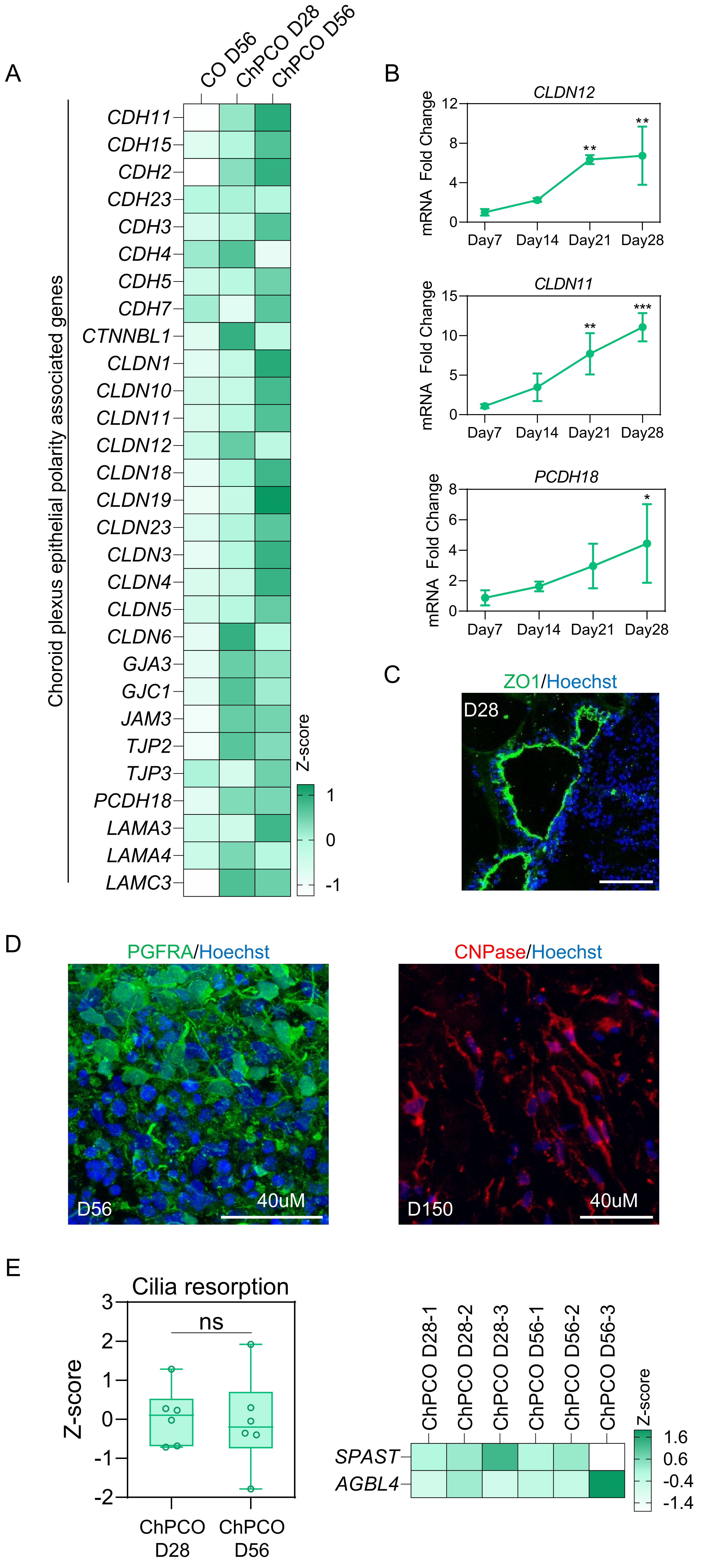
A
CO D56
ChPCO D28
ChPCO D56
Choroid plexus epithelial polarity associated genes
CDH11
CDH15
CDH2
CDH23
CDH3
CDH4
CDH5
CDH7
CTNNBL1
CLDN1
CLDN10
CLDN11
CLDN12
CLDN18
CLDN19
CLDN23
CLDN3
CLDN4
CLDN5
CLDN6
GJA3
GJC1
JAM3
TJP2
TJP3
PCDH18
LAMA3
LAMA4
LAMC3
Z-score
1
0
-1
B
CLDN12
CLDN11
PCDH18
mRNA Fold Change
Day7
Day14
Day21
Day28
C
ZO1/Hoechst
D28
D
PGFRA/Hoechst
D56
40uM
CNPase/Hoechst
D150
40uM
E
Cilia resorption
ns
Z-score
ChPCO D28
ChPCO D56
ChPCO D28-1
ChPCO D28-2
ChPCO D28-3
ChPCO D56-1
ChPCO D56-2
ChPCO D56-3
SPAST
AGBL4
1.6
0.6
-0.4
-1.4

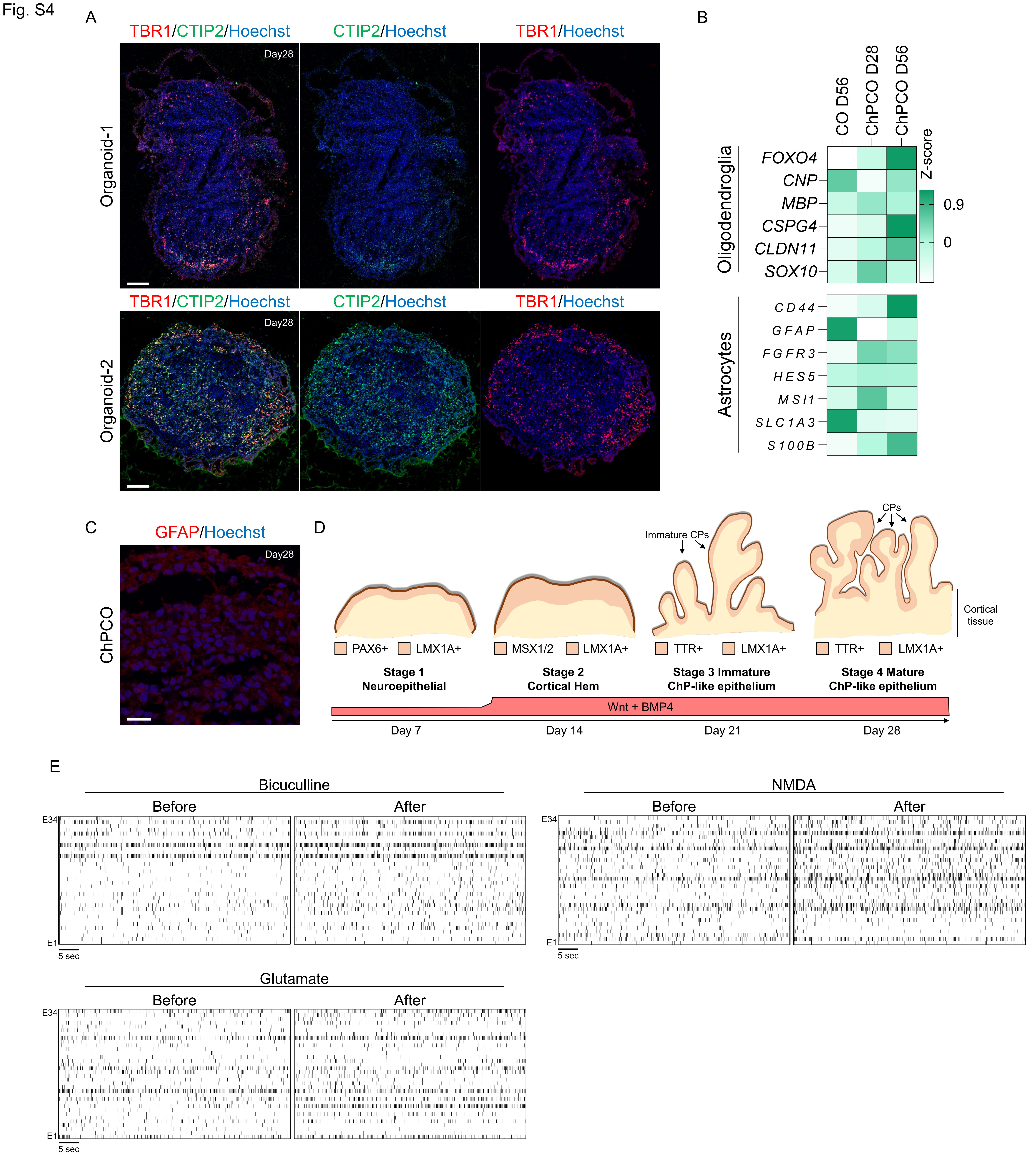
Fig. S4
A
TBR1/CTIP2/Hoechst
CTIP2/Hoechst
TBR1/Hoechst
Day28
Organoid-1
Organoid-2
B
CO D56
ChPCO D28
ChPCO D56
Oligodendroglia
FOXO4
CNP
MBP
CSPG4
CLDN11
SOX10
Z-score
0.9
0
Astrocytes
CD44
GFAP
FGFR3
HES5
MSI1
SLC1A3
S100B
C
GFAP/Hoechst
ChPCO
D
Immature CPs
CPs
Cortical tissue
PAX6+
LMX1A+
MSX1/2
TTR+
Stage 1 Neuroepithelial
Stage 2 Cortical Hem
Stage 3 Immature ChP-like epithelium
Stage 4 Mature ChP-like epithelium
Wnt + BMP4
Day 7
Day 14
Day 21
Day 28
E
Bicuculline
NMDA
Glutamate
Before
After
E34
E1
5 sec

Fig. S5

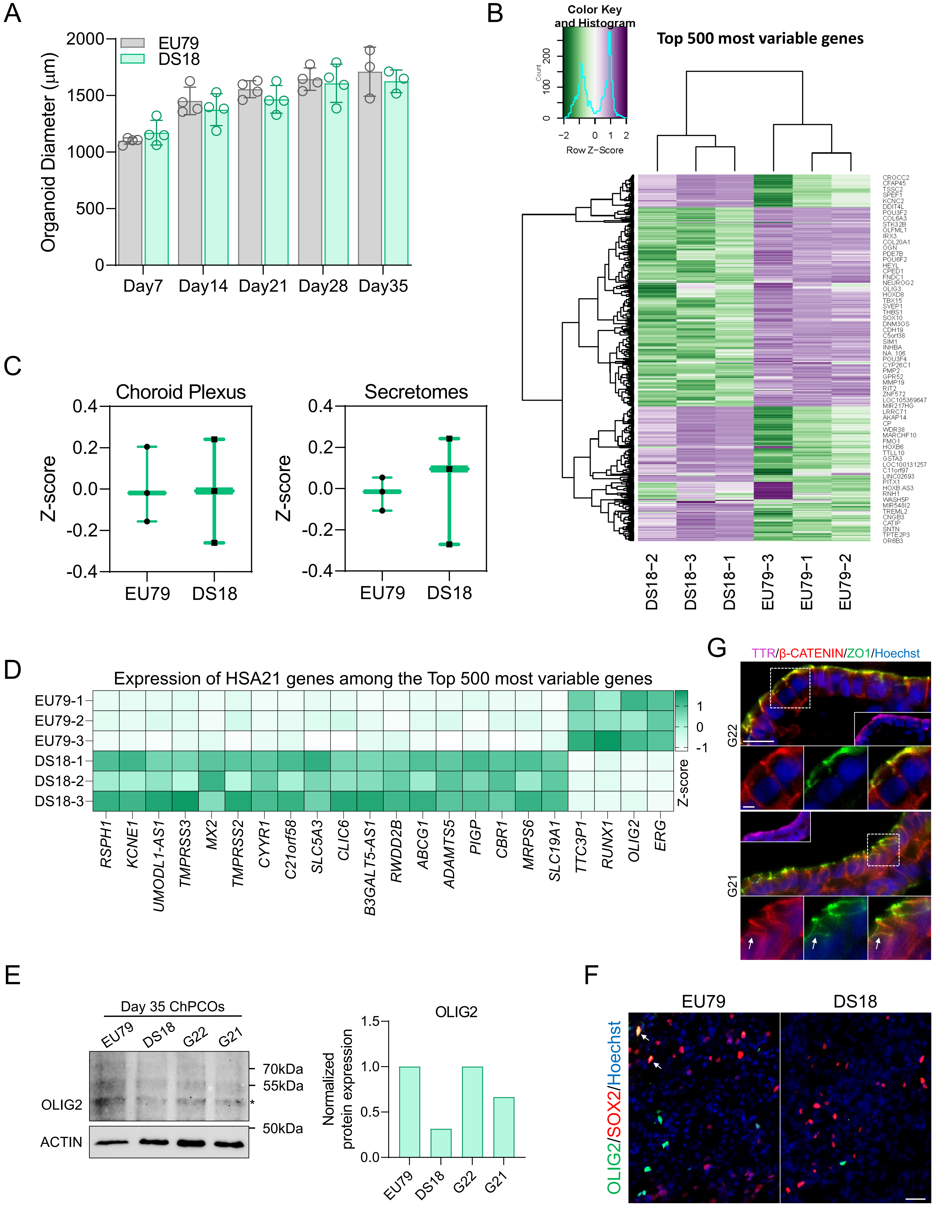
A
EU79
DS18
Organoid Diameter (µm)
2000
1500
1000
500
0
Day7
Day14
Day21
Day28
Day35
B
Color Key and Histogram
Count
Row Z-Score
Top 500 most variable genes
DS18-2
DS18-3
DS18-1
EU79-3
EU79-1
EU79-2
C
Choroid Plexus
Secretomes
Z-score
EU79
DS18
D
Expression of HSA21 genes among the Top 500 most variable genes
EU79-1
EU79-2
EU79-3
DS18-1
DS18-2
DS18-3
Z-score
RSPH1
KCNE1
UMODL1-AS1
TMPRSS3
MX2
TMPRSS2
CYYR1
C21orf58
SLC5A3
CLIC6
B3GALT5-AS1
RWDD2B
ABCG1
ADAMTS5
PIGP
CBR1
MRPS6
SLC19A1
TTC3P1
RUNX1
OLIG2
ERG
G
TTR/β-CATENIN/ZO1/Hoechst
G22
G21
E
Day 35 ChPCOs
EU79
DS18
G22
G21
70kDa
55kDa
50kDa
OLIG2
ACTIN
OLIG2
Normalized protein expression
1.5
1.0
0.5
0.0
F
EU79
DS18
OLIG2/SOX2/Hoechst

Fig. S6

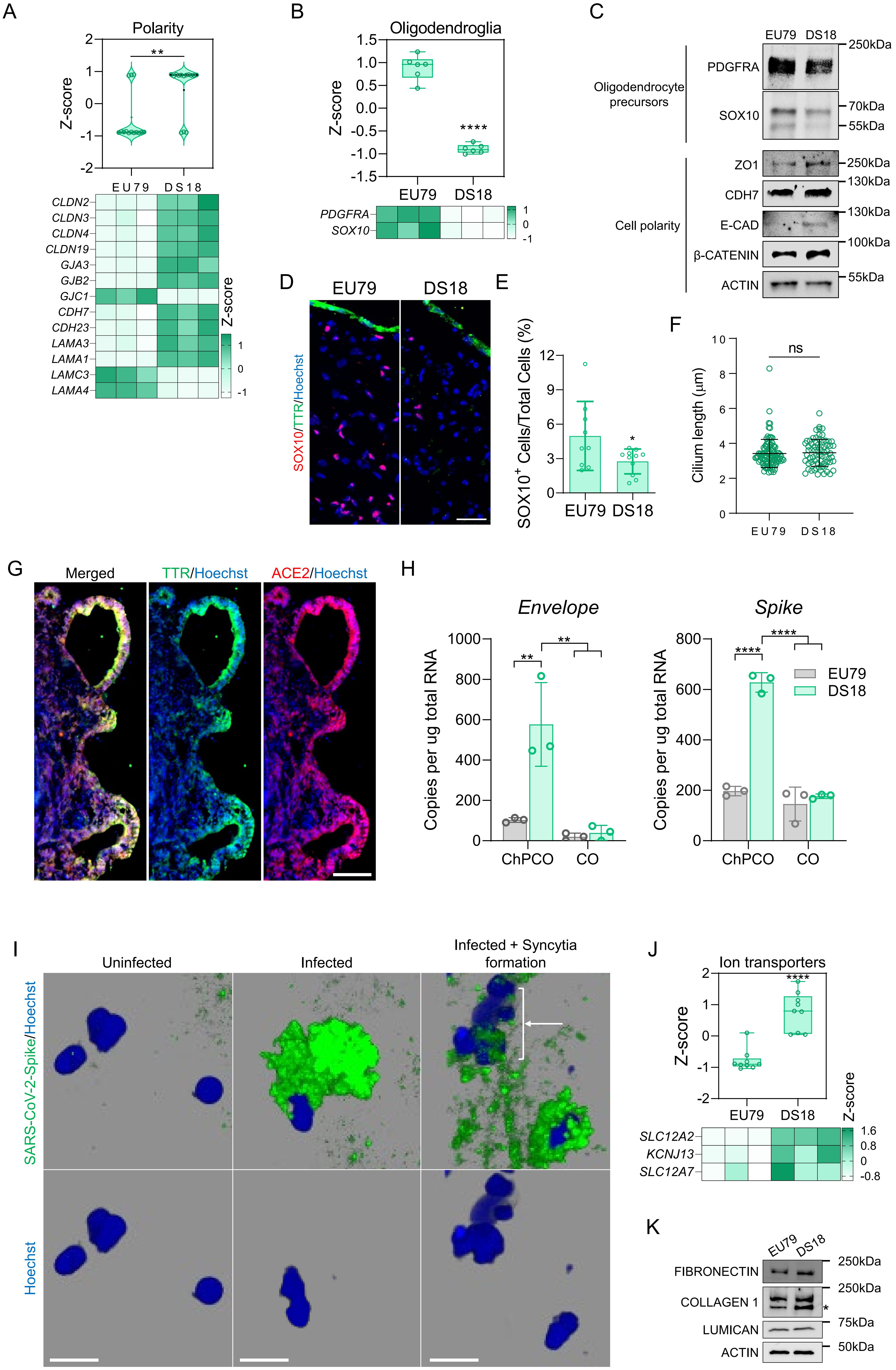
A
Polarity
Z-score
**
EU79
DS18
CLDN2
CLDN3
CLDN4
CLDN19
GJA3
GJB2
GJC1
CDH7
CDH23
LAMA3
LAMA1
LAMC3
LAMA4
B
Oligodendroglia
****
PDGFRA
SOX10
C
EU79
DS18
Oligodendrocyte precursors
PDGFRA
SOX10
250kDa
70kDa
55kDa
Cell polarity
ZO1
CDH7
E-CAD
β-CATENIN
ACTIN
130kDa
100kDa
D
EU79
DS18
SOX10/TTR/Hoechst
E
SOX10+ Cells/Total Cells (%)
F
ns
Cilium length (µm)
G
Merged
TTR/Hoechst
ACE2/Hoechst
H
Envelope
Spike
Copies per ug total RNA
ChPCO
CO
EU79
DS18
I
Uninfected
Infected
Infected + Syncytia formation
SARS-CoV-2-Spike/Hoechst
Hoechst
J
Ion transporters
SLC12A2
KCNJ13
SLC12A7
K
FIBRONECTIN
COLLAGEN 1
LUMICAN
ACTIN
75kDa
50kDa

Fig.S7

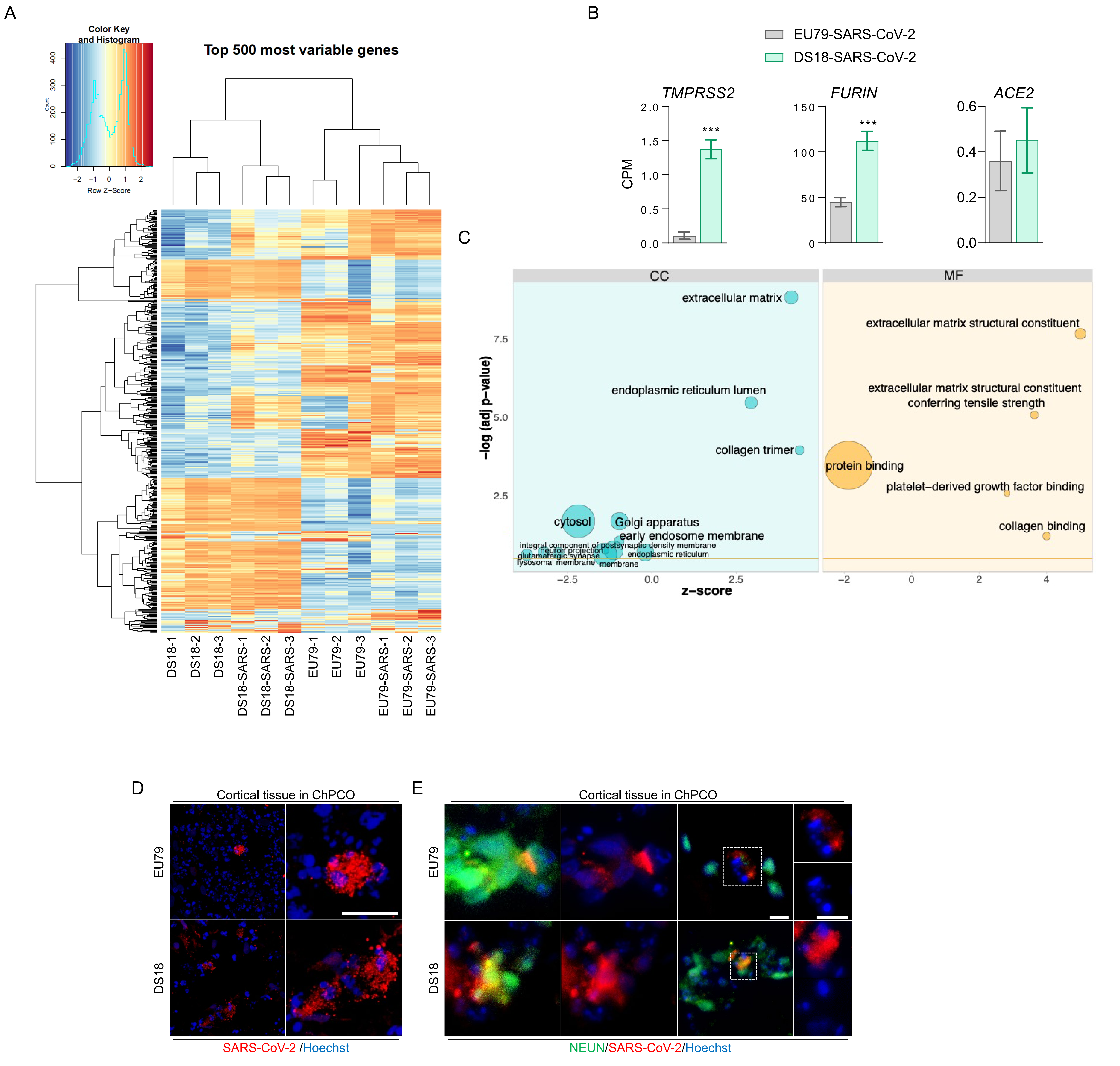
A
Color Key and Histogram
Row Z-Score
Top 500 most variable genes
DS18-1
DS18-2
DS18-3
DS18-SARS-1
DS18-SARS-2
DS18-SARS-3
EU79-1
EU79-2
EU79-3
EU79-SARS-1
EU79-SARS-2
EU79-SARS-3
B
EU79-SARS-CoV-2
DS18-SARS-CoV-2
TMPRSS2
FURIN
ACE2
CPM
C
CC
MF
extracellular matrix
endoplasmic reticulum lumen
collagen trimer
cytosol
Golgi apparatus
early endosome membrane
extracellular matrix structural constituent
extracellular matrix structural constituent conferring tensile strength
protein binding
platelet-derived growth factor binding
collagen binding
-log (adj p-value)
z-score
D
Cortical tissue in ChPCO
EU79
DS18
SARS-CoV-2 /Hoechst
E
Cortical tissue in ChPCO
NEUN/SARS-CoV-2/Hoechst

Fig.S8

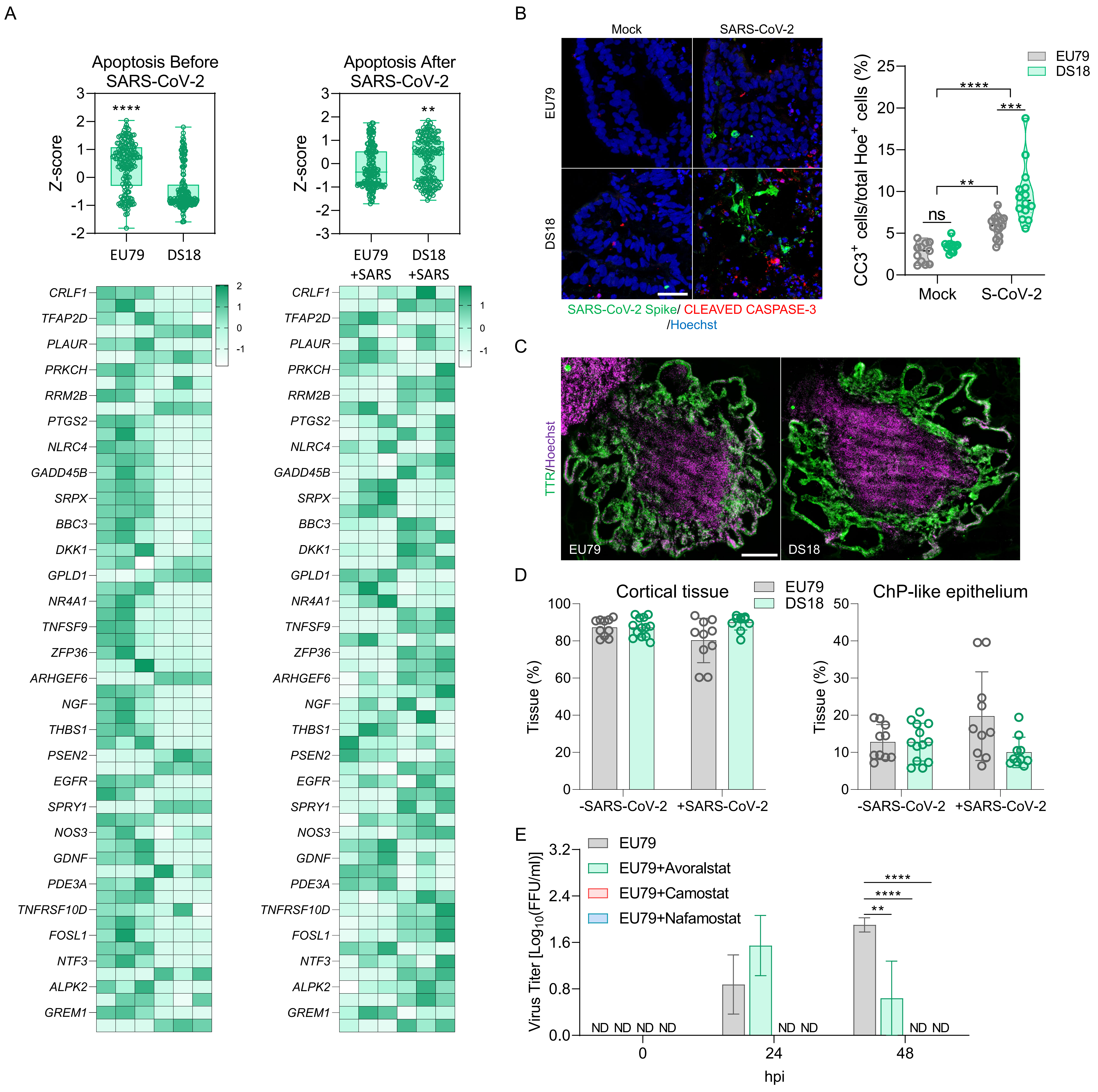
A
Apoptosis Before SARS-CoV-2
Apoptosis After SARS-CoV-2
Z-score
EU79
DS18
EU79 +SARS
DS18 +SARS
CRLF1
TFAP2D
PLAUR
PRKCH
RRM2B
PTGS2
NLRC4
GADD45B
SRPX
BBC3
DKK1
GPLD1
NR4A1
TNFSF9
ZFP36
ARHGEF6
NGF
THBS1
PSEN2
EGFR
SPRY1
NOS3
GDNF
PDE3A
TNFRSF10D
FOSL1
NTF3
ALPK2
GREM1
B
Mock
SARS-CoV-2
EU79
DS18
SARS-CoV-2 Spike/ CLEAVED CASPASE-3 /Hoechst
CC3+ cells/total Hoe+ cells (%)
ns
Mock
S-CoV-2
C
TTR/Hoechst
EU79
DS18
D
Cortical tissue
ChP-like epithelium
Tissue (%)
-SARS-CoV-2
+SARS-CoV-2
E
EU79
EU79+Avoralstat
EU79+Camostat
EU79+Nafamostat
Virus Titer [Log10(FFU/ml)]
ND
hpi

Fig.S9

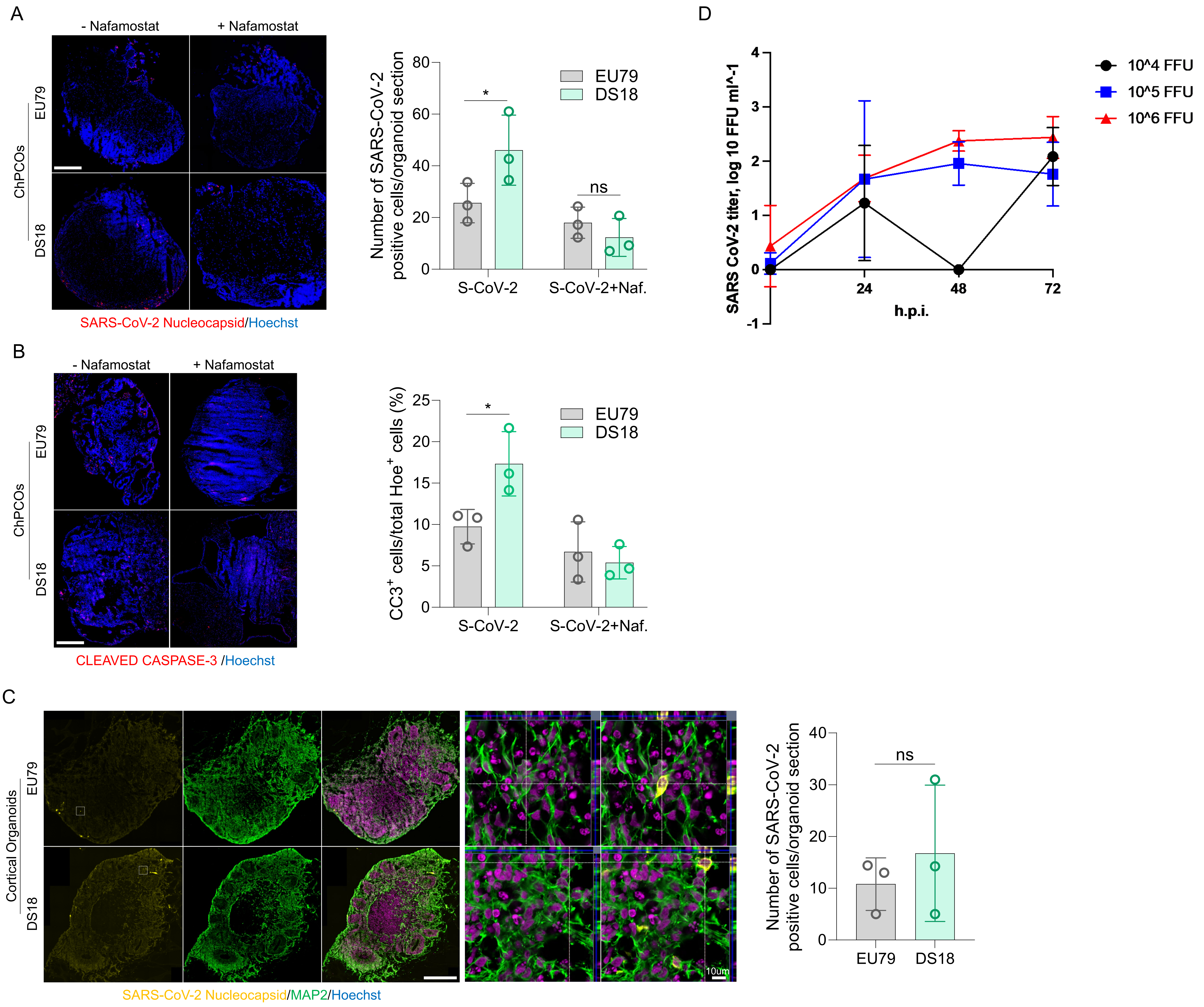
A
- Nafamostat
+ Nafamostat
ChPCOs
EU79
DS18
SARS-CoV-2 Nucleocapsid/Hoechst
Number of SARS-CoV-2 positive cells/organoid section
0
20
40
60
80
EU79
DS18
*
ns
S-CoV-2
S-CoV-2+Naf.
B
- Nafamostat
+ Nafamostat
ChPCOs
EU79
DS18
CLEAVED CASPASE-3 /Hoechst
CC3+ cells/total Hoe+ cells (%)
0
5
10
15
20
25
EU79
DS18
*
S-CoV-2
S-CoV-2+Naf.
C
Cortical Organoids
EU79
DS18
10um
SARS-CoV-2 Nucleocapsid/MAP2/Hoechst
Number of SARS-CoV-2 positive cells/organoid section
0
10
20
30
40
ns
EU79
DS18
D
SARS CoV-2 titer, log 10 FFU ml^-1
-1
0
1
2
3
4
24
48
72
h.p.i.
10^4 FFU
10^5 FFU
10^6 FFU

Supplemental Information

Figure S1. Characterization of Neuroectoderm Differentiation and Spheres Formation from hPSCs, related to Figure 1.

(**A**) Immunocytochemistry of human iPSCs stained with TRA-1-60 (Green), NANOG (Green), and SOX2 (Red). All cells were counterstained with Hoechst 33342 (Blue). Scale bar = 40 μm.

(**B**) Immunocytochemistry of human iPSCs-derived neuroectoderm labeled with NANOG (Green), SOX2 (Red), and NESTIN (Green) antibodies. All cells were counterstained with Hoechst 33342 (Blue). Scale bar = 20 μm.

(**C**) Wholemount immunostaining of hNEct spheroids at day 4 of FGF, CHIR99021, and BMP4 treatments stained with SOX2 (Red) and ZO1 (Green). The spheroid is counterstained with Hoechst 33342 (Blue). Scale bar = 100 μm.

(**D**) Wholemount immunostaining of hNEct spheroids at day 4 of FGF and different concentrations of BMP4 treatments stained with SOX2 (Green). The spheroids are counterstained with Hoechst 33342 (Blue). Scale bar = 50 μm. The right top graph represents the average intensity of SOX2 expression from center to periphery across FGF and BMP4 treated spheroids. Right bottom graph shows quantification of the percentage of cells expressing the neural stem cells marker (SOX2) relative to the total number of cells per sample. Data are presented as mean ± standard deviation. Number of independent experiments = 3. ****$P < 0.001$ via One Way ANOVA.

(**E**) Bright-field images of ChPCOs generated from hPSCs at day 28 of differentiation with and without embedding into ECM. Scale bar = 500 μm.

Figure S2. Reproducible Organization of ChP-like epithelia and Cortical Cells in ChPCOs Generated from Different hPSC lines, and Transcriptional Analysis of ChPCOs and COs related to Figure 1.

(A) Bright-field images of ChPCOs generated from H9-ESCs, G22-iPSCs and WTC-iPSCs line at day 28 of differentiation. Cross indicates the organoids without emerging thin epithelium. Scale bar = 500 μm.

(B) Representative images of sectioned ChPCOs immunostained with TTR (Green) and LMX1A (red) antibodies. All cells were counterstained with Hoechst 33342 (Blue). Scale bar = 200 μm. Dotted black lines indicate the cut bright-field ChPCOs shown in (A).

(C) Quantification of successful ChPCO generation at day 28 across different hPSC lines (H9-ESCs, G22-ESCs and WTC-iPSCs). Total number of experiments = 12; total number of analyzed organoids = 314. Data are presented as mean ± standard deviation. The number of organoids analyzed and the number of experiments from each cell line are summarized in Table S8.

(D) Heatmap expression of marker genes related to cortical hem, choroid plexus within the bulk RNA transcriptomes of ChPCOs at days 28 and 56 and COs at day 56. Values are shown as z-score.

(E) Heatmap expression of marker genes related to telencephalonic ChP within the bulk RNA transcriptomes of ChPCOs at days 28 and 56 and COs at day 56. Values are shown as z-score.

(F) Hierarchical clustering of gene expression profiles of ChPCOs at day 28 and 56, COs at day 56 and human ChP tissue (obtained from 44-70 years old donors (*104*)) based on the expression of multiple ChP markers. The values are read counts normalized to library sizes, composition bias and log+1-transformed.

Figure S3. Analysis of Tight Junction and Neural Cells During the Development of ChPCOs, related to Figures 2&3.

(**A**) Heatmap expression of marker genes related to apicobasal polarity within the bulk RNA transcriptomes of ChPCOs at days 28 and 56 and COs at day 56. Values are shown as z-score.
(**B**) qRT-PCR of marker genes related to apicobasal polarity (*CLDN12, CLDN11,* and *PCDH18*) in ChPCOs. All values were normalized to GAPDH levels of their respective samples and expressed relative to Day 7 values to obtain the fold change. Data are shown as the mean ± standard deviation; **p < 0.01, ***p < 0.001, ****p < 0.0001 via one-way ANOVA. Number of independent experiments = 3.
(**C**) Analysis of immunostaining of sections ChPCOs at day 28 showing tight junction ZO1 (Green) protein expression in the epithelial cells of ChPs. Section was counterstained with Hoechst 33342 (Blue). Scale bar = 72 µm.
(**D**) Analysis of immunostaining of sections ChPCOs at days 56 and 150 showing oligodendrocyte progenitors marked with PDGFRA (Green), and mature oligodendrocytes marked with CNPase (Red) proteins. Section was counterstained with Hoechst 33342 (Blue). Scale bar = 40 µm.
(**E**) Box blot showing distribution of marker genes (listed below in the heatmap) associated with cilia resorption from bulk RNA-seq of ChPCOs at days 28 and 56. Data are presented as minimum to maximum, with individual dots represent individual replicate of genes listed below. Right panel is heatmap expression of marker genes related to cilia resorption. Values are shown as z-score.

Figure S4. Validation of Cortical Neurons Emerging in ChPCOs Generated from Different Batches, related to Figure 3.

(**A**) Analysis of immunostained ChPCO sections at day 28 obtained from different batches showing cortical neurons in layer VI marked by TBR1 (Red) and layer V marked by CTIP2 (Green) proteins. All sections were counterstained with Hoechst 33342 (Blue). Scale bar = 122 µm.

(**B**) Heatmap expression of marker genes related to oligodendroglia and astrocytes within the bulk RNA transcriptomes of ChPCOs at days 28 and 56 and COs at day 56. Values are shown as z-score.

(**C**) Analysis of ChPCOs sections at day 28 immunostained with astrocyte marker GFAP (Red) protein. Section was counterstained with Hoechst 33342 (Blue). Scale bar = 20 µm.

(**D**) Schematic diagram summarizing the developing structure of ChPCOs at different days of development showing the specified the two domains including ChPs, and the cortical tissue ~~cortex~~ and the culture conditions used.

(**E**) Representative images of raster plots of a single MEA show the intensity of spiking activity before and after Bicuculline, NMDA, Glutamate treatments. Raster plots are shown in 1 min segments. E is electrode.

Figure S5. Analysis of Euploid and Trisomy 21 ChPCOs Characteristics, related to Figure 4.

(**A**) Graph showing the growth (average diameter) of ChPCOs in euploid (EU79) and trisomy 21 (DS18) at different stages of *in vitro* culture. Data are presented as mean ± standard deviation (n = 4).

(**B**) Hierarchical clustering of the transcriptomes of DS(DS18) and euploid (EU79) ChPCOs based on the expression of top 500 most variable genes.

(**C**) Box blots showing distribution of differentially expression genes associated with choroid plexus and secretomes in euploid (EU79) and trisomy 21 (DS18) ChPCOs at day 28 obtained from bulk RNA-seq. Data are presented as minimum to maximum, with notches are centered on the median. Values are shown as z-score.

(**D**) Heatmap expression of HSA21 genes among the top 500 most variable genes within the bulk RNA transcriptomes of euploid (EU79) and DS (DS18) ChPCOs. Values are shown as z-score.

(**E**) Western blots showing the protein levels of OLIG2 in euploids (EU79 and G22) and DS (DS18 and G21) ChPCOs at day 35. Actin was used for normalization. All blots derived from the same experiment and processed in parallel. The right graph shows the quantification of normalized OLIG2 level obtained from blots.

(**F**) Analysis of euploid (EU79) and DS (DS18) ChPCO sections at day 35 immunostained with OLIG2 (Green) and SOX2 (Red) antibodies. All sections were counterstained with Hoechst 33342 (Blue). Scale bar = 20 µm.

(**G**) Immunostaining of euploid (G22) and DS (G21) ChPCOs at day 56 for β-catenin (Red) and ZO1 (Green) and TTR (Magenta). All sections were counterstained with Hoechst 33342 (Blue). Scale bar = 25 µm. Dotted white boxes denote areas that are magnified and where the two fluorescent channels. Scale bar = 10 µm.

Figure S6. Analysis of Oligodendroglia, Apicobasal Polarity, and Electrophysiological Activity Characteristics of Euploid and Trisomy 21 ChPCOs, related to Figures 4, 5.

(**A**) Violin blot showing distribution of marker genes (listed below in the heatmap) associated with apicobasal polarity obtained from bulk RNA-seq of ChPCOs at day 28 derived from euploid (EU79) and DS (DS18) lines. Data are presented as minimum to maximum, with individual dots representing individual replicate of genes listed below. $^{**}p < 0.01$ via Student's t-test. Below heatmap expression of marker genes related to apicobasal polarity. Values are shown as z-score.

(**B**) Box blot showing distribution of marker genes (listed below in the heatmap) associated with oligodendroglia obtained from bulk RNA-seq of ChPCOs at day 28 derived from euploid (EU79) and DS (DS18) lines. Data are presented as minimum to maximum, with individual dots representing individual replicate of genes listed below. $^{****}p < 0.0001$ via Student's t-test. Below heatmap expression of marker genes related to oligodendroglia. Values are shown as z-score.

(**C**) Western blots showing the protein levels of oligodendrocyte precursors (PDGFRA, SOX10) and cell polarity (ZO1, CDH7, E-CAD, β-CATENIN) in euploid (EU79) and DS (DS18) ChPCOs at day 49. Actin was used for normalization. All blots derived from the same experiment and processed in parallel.

(**D**) Analysis of euploid (EU79) and DS (DS18) ChPCO sections at day 56 immunostained SOX10 (Red) and the ChP marker TTR (Green) antibodies. All sections were counterstained with Hoechst 33342 (Blue). Scale bar = 30 µm.

(**E**) Bar graph showing the percentage of SOX10 expressing cells in euploid (EU79) and DS (DS18) ChPCOs at day 56. Data are presented as mean ± standard deviation. $^{*}p < 0.05$ indicates statistical significance via Student's t-test. Number of independent experiments = 3.

(**F**) Quantification of cilia length in ChPCOs derived from euploid (EU79) and DS (DS18) lines at day 56 of differentiation. Data are presented as mean ± standard deviation; ns is not significant via Student's t-test. Number of independent experiments = 3. Individual dots represent a cilium length.

(**G**) Representative image of trisomy 21-derived ChPCOs at day 35 immunostained with ACE2 (Red), and TTR (Green). Section was counterstained with Hoechst 33342 (Blue). Scale bar = 100 µm.

(**H**) Bar graphs showing the amount of SARS-CoV-2 related genes *Envelop* and *Spike* in euploid (EU79) and trisomy 21 (DS18) ChPCOs at day 28. Data are presented as mean ±

standard deviation. $**p < 0.01$, $****p < 0.0001$ via 2-Way ANOVA. Number of independent experiments = 3.

(**I**) High-resolution imaging and 3D reconstruction of cells with and without SARS-CoV-2 (Green) infection with maximum Z-planes projection to highlight multiple nuclei. Section was counterstained with Hoechst 33342 (Blue). White arrow indicates syncytia formation. Scale bar = 10 μm.

(**J**) Box blot showing distribution of marker genes (listed below in the heatmap) associated with ion transport obtained from bulk RNA-seq of ChPCOs at day 28 derived from euploid (EU79) and DS (DS18) lines. Data are presented as minimum to maximum, with individual dots represent individual replicate of genes listed below. $****p < 0.0001$ via Student's t-test. Below heatmap expression of marker genes related to ion transport. Values are shown as z-score.

(**K**) Western blots showing the protein levels of ECM proteins including FIBRONECTIN, COLLAGEN 1 AND LUMICAN euploid (EU79) and DS (DS18) ChPCOs at day 49. ACTIN was used for normalization. All blots derived from the same experiment and processed in parallel.

Figure S7. Expression of SARS-CoV-2 related genes in Organoids, related to Figure 6.

(**A**) Hierarchical clustering heat map of the top 500 most variable genes expressed in DS18 and EU79 ChPCOs that were infected with SARS-CoV-2 or uninfected.
(**B**) Expression of host genes required for SARS-CoV-2 infection in DS18 and EU79 ChPCOs. The values represent normalized read counts expressed as counts per million (cpm). The graphs show the mean values from 3 biological replicates with error bars indicating SDs. The statistical analysis was performed by Student's t-test. ***$P<0.001$.
(**C**) Gene ontology (GO) enrichment analysis of differentially expressed genes identified in Figure 6F. Z-scores indicate the cumulative increase or decrease in expression of the genes associated with each term. The size of the bubbles is proportional to the number of DEGs associated with respective GO term. MF – molecular function, CC – cellular component.

(**D-E**) Representative confocal images of day 31 ChPCOs derived from euploid (EU79) and DS (DS18) immunostained with NEUN (Green) and SARS-CoV-2 spike (Red) after SARS-CoV-2 (10^6 FFUs). All sections were counterstained with Hoechst 33342 (Blue). Scale bar = 10 μm.

Figure S8. Analysis of Apoptotic Process in SARS-CoV-2 infected ChPCOs, related to Figure 5&7.

(**A**) Violin blot showing distribution of marker genes (listed below in the heatmap) associated with apoptosis obtained from bulk RNA-seq of day 31 ChPCOs before and after SARS-CoV-2 infection derived from euploid (EU79) and DS (DS18) lines. Data are presented as minimum to maximum, with individual dots representing individual replicate of genes listed below. $^{**}p < 0.01$; $^{****}p < 0.0001$ via Student's t-test. Below heatmap expression of marker genes related to apoptosis. Values are shown as z-score.

(**B**) Analysis of sections of euploid (EU79) and DS (DS18) day 31 ChPCO infected with Mock and SARS-CoV-2 over 72 hpi immunostained with Cleaved caspase-3 (Red) and SARS-CoV-2 spike (Green) antibodies. All sections were counterstained with Hoechst 33342 (Blue). Scale bar = 35 μm. Below violin graph showing the percentage of cleaved caspase 3 positive cells in ChPCOs derived from euploid (EU79) and DS (DS18) infected with the Mock and SARS-CoV-2 virus. $^{**}p < 0.01$; $^{***}p < 0.001$; $^{****}p < 0.0001$ via 2-Way ANOVA.

(**C**) Representative image of euploid and DS ChPCOs at day 31 immunostained with TTR (Green). The section was counterstained with Hoechst 33342 (Purple). Scale bar = 200 μm.

(**D**) Quantification of choroid plexus-like epithelium and cortical tissues compartments in at least 3 different euploid (EU79) and DS (DS18) hiPSC derived ChPCOs at day 31 with and without SARS-CoV-2 infection. Individual dots represent a single organoid.

(**E**) Quantification of viral titers from ChPCOs culture supernatants of euploid (EU79), DS (DS18), and EU79 treated with 100uM avoralstat or camostat or nafamostat after SARS-CoV-2 (10^6 FFUs) infection of day 28 ChPCOs at 0, 24, and 48 hpi. Data are presented as mean ± SEM. N = 3 biological replicates consisting of 3 organoids each; $^{**}p < 0.01$; $^{****}p < 0.0001$ via One-Way ANOVA.

Figure S9. Analysis of SARS-CoV-2 infection Apoptotic Process in treated SARS-CoV-2 infected ChPCOs with Nafamostat, related to Figure 5&7.

(**A**) Representative confocal images of day 31 ChPCOs derived from euploid (EU79) and DS (DS18) treated with and without Nafamostat and immunostained with SARS-CoV-2 Nucleocapsid (Red) after SARS-CoV-2 (10^6 FFUs). All sections were counterstained with Hoechst 33342 (Blue). Scale bar = 400 µm. Bar graph showing the frequency of SARS-CoV-2-positive cells in ChPCOs derived from euploid (EU79) and DS (DS18) with and without Nafamostat treatment. Data are presented as mean ± SD. *$p < 0.05$ via via 2-Way ANOVA. 36 ChPCOs sections from three (n = 3) independent samples were examined.
(**B**) Representative confocal images of day 31 ChPCOs derived from euploid (EU79) and DS (DS18) treated with and without Nafamostat and immunostained with CLEAVED-CASPASE-3 (Red) after SARS-CoV-2 (10^6 FFUs). All sections were counterstained with Hoechst 33342 (Blue). Scale bar = 400 µm. Bar graph showing the frequency of SARS-CoV-2-positive cells in ChPCOs derived from euploid (EU79) and DS (DS18) with and without Nafamostat treatment. Data are presented as mean ± SD. *$p < 0.05$ via 2-Way ANOVA. 36 ChPCOs sections three independent samples were examined.
(**C**) Representative confocal images of day 31 COs derived from euploid (EU79) and DS (DS18) treated with and without Nafamostat and immunostained with MAP2 (Green), and SARS-CoV-2 Nucleocapsid (Yellow) after SARS-CoV-2 (10^6 FFUs). Scale bar = 400 µm. Zoomed images are Orthogonal projections (x/y, x/z, and y/z) of confocal z-stacks. All sections were counterstained with Hoechst 33342 (Purple). Scale bar = 10 µm. Bar graph showing the frequency of SARS-CoV-2-positive cells in COs derived from euploid (EU79) and DS (DS18). Data are presented as mean ± SD. 18 ChPCOs from three independent samples were examined.
(**D**) Replication of SARS-CoV-2 in euploids hCOs inoculated with different doses of the virus. Organoids were inoculated with 10^4, 10^5 and 10^6 FFU of SARS-CoV-2 and viral titer were determined by immunofluorescent foci-forming assay at the indicated time points. Data are presented as mean ± SD. N = 3 biological replicates consisting of 3 organoids each.

Table S1. Differentially expressed genes in DS18 vs EU79 CPCOs.

Table S2. Differentially expressed genes in DS18 ChPCOs infected with SARS-CoV-2 compared to mock.

Table S3. Differentially expressed genes in SARS-CoV-2-infected EU79 ChPCOs compared to mock.

Table S4. Differentially expressed genes in SARS-CoV-2-infected DS18 compared to SARS-CoV-infected EU79 ChPCOs.

Table S5. List of genes associated with apoptosis across DS18 and euploid groups before and after SARS-CoV-2 infection.

Table S6. List of Primer Sequences used for RT-PCR (5′−3′ orientation).

Table S7. List of Antibodies used for immunohistochemistry.

Table S8. Table of total number of experiments and organoids analyzed from different cell lines, related to Figure 1, Figure 2, Figure 3, Figure 4, Figure 7, and Figure S3
